# In Vivo Characterization of the *dia* Biosynthetic Gene Cluster Reveals Diaporthinic Acid as Its Main Product

**DOI:** 10.3390/jof12060402

**Published:** 2026-06-01

**Authors:** Isabella Burger, Simon Leonhartsberger, Kathrin Peikert, Lukas Fourtis, Polina Atanasova, Lara T. S. Kramer, Richard Fried, Christian Stanetty, Florian Rudroff, Ruth Birner-Gruenberger, Robert L. Mach, Astrid R. Mach-Aigner, Matthias Schittmayer, Christian Zimmermann

**Affiliations:** 1 Institute of Chemical Technologies and Analytics, TU Wien, 1060 Vienna, Austria; isabella.burger@univie.ac.at (I.B.); ruth.birner-gruenberger@tuwien.ac.at (R.B.-G.); 2 Department of Analytical Chemistry, Faculty of Chemistry, University of Vienna, 1090 Vienna, Austria; 3 Institute of Applied Synthetic Chemistry, TU Wien, 1060 Vienna, Austria; simon.leonhartsberger@tuwien.ac.at (S.L.); richard.fried@tuwien.ac.at (R.F.); christian.stanetty@tuwien.ac.at (C.S.); florian.rudroff@tuwien.ac.at (F.R.); 4 Institute of Chemical, Environmental and Bioscience Engineering, TU Wien, 1060 Vienna, Austriarobert.mach@tuwien.ac.at (R.L.M.); astrid.mach-aigner@tuwien.ac.at (A.R.M.-A.)

**Keywords:** ascomycetes, *Aspergillus oryzae*, gene expression, secondary metabolism, transcription factor, regulation

## Abstract

The biosynthetic gene cluster (BGC) responsible for producing diaporthinic acid has remained genetically unassigned despite repeated isolation of this metabolite from several fungal species. In this study, we activated the *dia* BGC in *Trichoderma reesei* by overexpressing the cluster-associated zinc cluster protein DiaR1 to identify the BGC’s in vivo metabolic output and reconstruct the corresponding biosynthetic pathway. Metabolite production was analyzed by HPLC-MS/MS, and the major product was isolated and structurally confirmed by NMR spectroscopy. Individual genes of the *dia* cluster were deleted in the activated background to assess their functional roles, and transcript levels were quantified by RT-qPCR. Activation of the cluster resulted in the predominant accumulation of diaporthinic acid, accompanied by several related isocoumarin derivatives, while antibacterial and antifungal assays showed no detectable activity of diaporthinic acid under the tested conditions. Deletion analyses demonstrated that the polyketide synthase Dia1, the bifunctional halogenase/methyltransferase Dia5, and the FAD-dependent oxidoreductase Dia4 are essential for diaporthinic acid formation, whereas Dia2 and Dia3 are dispensable in vivo despite the previously proposed roles of their *Aspergillus oryzae* homologs based on in vitro studies. On the basis of intermediate accumulation patterns, we propose that Dia4 catalyzes the oxidation of dichlorodiaporthin to diaporthinic acid. Together, these results genetically link diaporthinic acid to the *dia* BGC and refine the previously proposed biosynthetic model derived from *A. oryzae*.

## 1. Introduction

**Figure 1:**
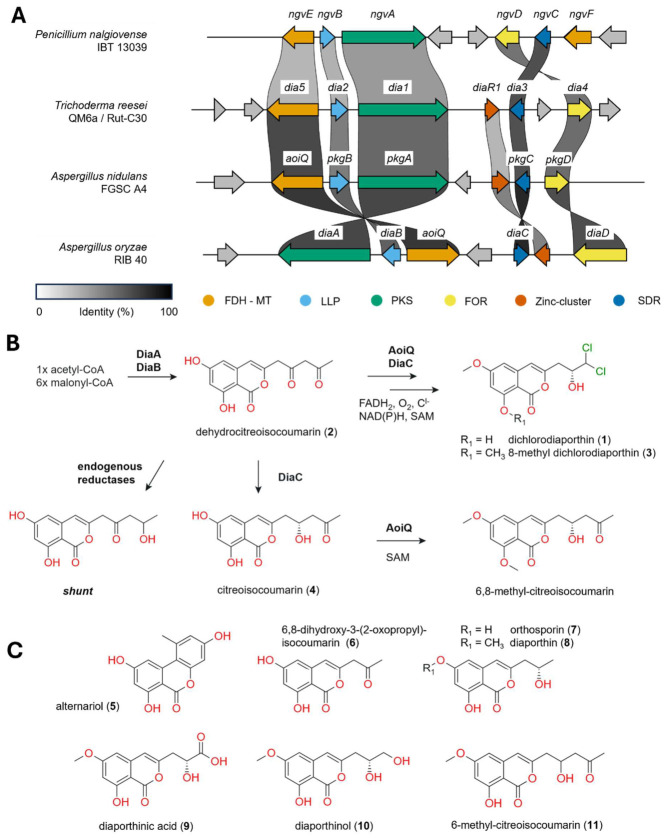
(**A**) The *dia* BGC and its homologs from the indicated fungal strain were compared and visualized using the cblaster and the clinker tool [9]. FDH-MT, flavin-dependent halogenase-methyltransferase; LLP, beta-lactamase-like protein; PKS, polyketide synthase; FOR, FAD-dependent oxidoreductase; Zinc cluster (protein); SDR, short chain dehydrogenase/reductase. (**B**) Graphical summary of the main findings within the biosynthetic pathway towards 8-methyl dichlorodiaporthin according to Liu et al. [6]. (**C**) Other diaporthin-related reported metabolites.

Fungal biosynthetic gene clusters (BGCs) encoding halogenated polyketides are of particular interest because halogenation can strongly influence biological activity and chemical diversity [1,2,3,4]. Among these, the *dia* cluster is notable for the presence of an unusual bifunctional enzyme, AoiQ, which combines a halogenase domain and a methyltransferase domain [5]. Chankhamjon et al. first described that this enzyme is essential for the formation of dichlorodiaporthin (**1**) in *Aspergillus oryzae* [5]. The *dia* BGC also contains the genes for a non-reducing polyketide synthase (PKS, *diaA*), a β-lactamase-like enzyme (*diaB*), a short-chain dehydrogenase/reductase (SDR, *diaC*), and a flavin-dependent monooxygenase (FMO, *diaD*) [5]. Homologs of the *dia* BGC are found in several other fungi (Figure 1A and Table 1), including *A. nidulans*, *Trichoderma harzianum*, *T. virens*, and *T. reesei*. Notably, the homologous BGC in *Penicillium nalgiovense* contains two distinct genes encoding separate halogenase and methyltransferase (Figure 1A) [6].

In 2022, Liu et al. studied parts of the *A. oryzae dia* BGC biosynthesis by heterologous expression of the enzymes DiaA-C and AoiQ in *Saccharomyces cerevisiae* and by several in vitro assays with AioQ and DiaC produced in *Escherichia coli* [6]. Based on their results, Liu et al. found that the heterologous expression results in the formation of dichlorodiaporthin (**1**) and proposed the following biosynthetic pathway (Figure 1B). DiaA and DiaB are responsible for the formation of dehydrocitreoisocoumarin (**2**). As the PKS does not contain an enoyl-reductase domain, which is normally involved in the release of the polyketide, the β-lactamase-like enzyme is suggested to take over this role. The model of Liu et al. describes DiaC and AoiQ acting simultaneously, resulting in the formation of 8-methyl dichlorodiaporthin (**3**). They also reported on several intermediates and shunt products, including citreoisocoumarin (**4**). Notably, the *A. oryzae* AoiQ appears to have the unique ability to catalyze the *O*-8 methylation of (**2**); substitutions with the AoiQ homologs from *P. nalgiovense*, *A. nidulans*, or *T. harzianum* in the yeast assays resulted in the formation of dichlorodiaporthin (**1**) instead of (**3**) [6]. Importantly, this study did not include the DiaD enzyme.

In another study, the DiaA and DiaB homologs of *A. nidulans* (PkgA, ANID_07071.1 and PkgB, ANID_07070.1) were overexpressed in the native host [7]. Ahuja et al. reported on the detection of compounds with masses corresponding to citreoisocoumarin (**4**) and alternariol (**5**) (Figure 1C) [7]. Liu et al. also found small amounts of (**4**), which was attributed to the activity of DiaC on (**2**), but they did not report on the formation of (**5**) [6].

Notably, *A. oryzae* possesses a second BGC, which is somewhat similar to its *dia* BGC—the *aoi* BGC. It contains a PKS, an oxygenase, and a methyltransferase [8]. This BGC was activated previously by a transcription factor overexpression strategy [8]. Nakazawa et al. suggested that the PKS AoiG synthesized 6,8-dihydroxy-3-(2-oxopropyl)-isocoumarin (**6**), which was reduced by the oxidoreductase AoiI, yielding orthosporin (**7**). Orthosporin (**7**) was suggested to be further methylated by AoiF, resulting in diaporthin (**8**), based on elevated transcript levels for these enzymes in the activated strain (Figure 1C). Later, Chankhamjon et al. suggested that the methylation of (**7**) might be catalyzed by AoiQ based on in vitro assays [5]. However, (**1**) and (**8**) are considered to be the products of two distinct BGCs in *A. oryzae* [5,6].

Independent of these pathway assignments in *A. oryzae*, several studies have reported the co-occurrence of multiple diaporthin-related metabolites in other fungal species. In *P. nalgiovense*, (**1**) was detected together with diaporthinic acid (**9**) and diaporthinol (**10**) in three isolates (IBT 12679, IBT 13296, and IBT 13330) [10]. Likewise, in *D. pomorum*, (**1**), (**4**), (**8**), (**9**), (**10**), and 6-methyl-citreoisocoumarin (**11**) were consistently detected in all 27 strains analyzed [11]. The genomes of the *P. nalgiovense* and *D. pomorum* strains used in these studies are not available, but we found homologs of the *dia* BGCs in the genomes of sequenced strains using the CompArative GEne Cluster analysis toolbox, https://cagecat.bioinformatics.nl/ accessed 15 November 2024 [9]. Exemplarily, the BGC of the *P. nalgiovense* strain IBT 13039 is included in Figure 1. Homologs of *T. reesei* DiaR1 and Dia1-5 are also found in the genome of the Canadian isolate *D. pomorum* M27-16 on scaffold 121 (GenBank: JAKJXN020000121) (Table S1).

Importantly, the flavin-dependent monooxygenase DiaD or its homologs have not yet been functionally characterized, and their role in the pathway remains unknown. Moreover, the current pathway model for the *dia* BGC is based on heterologous expression and in vitro enzymatic assays [6], while direct genetic evidence from an intact fungal host is limited to an upregulation of only *pkgA* and *pkgB* in *A. nidulans* [8]. Consequently, neither the complete in vivo biosynthetic sequence nor the final metabolic output of the *dia* cluster has been experimentally established in any fungus to date. In particular, although (**9**) has been repeatedly isolated from *dia* cluster–harboring fungi, it has not been genetically linked to this BGC.

In this study, we activated the *dia* BGC in *T. reesei* using a transcription factor overexpression strategy. We isolated and identified the main product of the *dia* BGC, diaporthinic acid (**9**), via NMR and tested it for antibacterial and antifungal properties. We further deleted all *dia* genes individually in the activated strain and performed HPLC-MS/MS analyses to scrutinize the in vivo biosynthetic pathway and compare it to previous studies. Additionally, we measured the transcript levels of the genes in all strains to gain insights into potential additional regulatory mechanisms. Finally, we deleted the gene *dia3* also in the wild-type background to test its influence on the physiology of *T. reesei* within and outside of the *dia* biosynthetic pathway.

## 2. Materials and Methods

### 2.1. Fungal Strains and Cultivation Conditions

All *T. reesei* strains (Table 2) were maintained on malt extract (MEX) agar plates (30 g L^−1^ malt extract, 1 g L^−1^ peptone, 15 g L^−1^ agar) at 30 °C. Uridine and hygromycin were added, if necessary, to the final concentrations of 5 mM and 75 µg mL^−1^, respectively. The strains were cultivated in Mandels-Andreotti medium [12] (KH_2_PO_4_ 2 g, (NH_4_)_2_SO_4_ 1.4 g, Urea 0.3 g, FeSO_4_·7H_2_O 0.005 g, MnSO_4_·H_2_O 0.0016 g, ZnSO_4_·7H_2_O 0.0014 g, CoCl_2_ 0.002 g, MgSO_4_·7H_2_O 0.3 g, CaCl_2_ 0.3 g, peptone 0.75 g) with 10 g L^−1^ glycerol as carbon source at 30 °C at 180 rpm in an orbital shaker. Samples were taken after 48 h for RNA extraction and biomass determination or 72 h for metabolite extraction and analysis. The mycelium for RNA extraction was stored at −80 °C. For biomass determination, the mycelium was dried at 80 °C overnight until completely dry and weighed on a precision scale. The culture supernatants for metabolite analyses were stored at –20 °C.

### 2.2. Fungal Transformation

*T. reesei* was transformed using a polyethylene glycol (PEG)-mediated transformation of protoplasts. Spores of the recipient strain were plated on cellophane sheets on MEX plates and incubated overnight at 30 °C. Mycelium was scraped, transferred into 15 mL Buffer A (1.2 M sorbitol, 100 mM KH_2_PO_4_, pH 5.6) with 600 mg Vinotaste Pro (Novozymes, Bagsværd, Denmark) and 0.5 mg chitinase (Sigma-Aldrich, St. Louis, MO, USA), and incubated at 60 rpm and 30 °C for 2–3 h until protoplasts were released. The suspension was filtered (70 µm sieve), chilled on ice, filled up to 40 mL with ice-cold 1.2 M sorbitol and centrifuged at 2500× *g* at 4 °C for 10 min. Then, it was washed in 30 mL ice-cold 1.2 M sorbitol. The protoplasts were resuspended in 1 mL Buffer B (1 M sorbitol, 25 mM CaCl_2_, 10 mM Tris-HCl, pH 7.5).

For transformation, DNA (20 µg linearized plasmid or 5 µg fusion PCR products) was mixed with 100 µL protoplast suspension and 100 µL 20% PEG (mixture of 6.7 mL Buffer B and 3.3 mL “60% PEG” (60 g PEG 4000, 1 mL 1 M Tris-HCl pH 7.5, 1 mL 1 M CaCl2, 38 mL ddH2O)). After 30 min on ice, 60% PEG was added incrementally, followed by incubation at room temperature for 20 min. Buffer C (1 M sorbitol, 10 mM Tris-HCl, pH 7.5) was added gradually, and the mixture was diluted to 50 mL with warm selection medium (containing 1 M sucrose) before plating. Plates were incubated at 30 °C under light for up to a week, and colonies were purified by spore streaking on selection plates containing 0.1% (*v*/*v*) Igepal CA-630.

### 2.3. Strain Construction Strategies

The sequences of all used primers are given in Supplemental Material File S8. For the construction of *T. reesei* OEdiaR1, the coding region of *diaR1* (protein ID 111742) was amplified with the primers 111742_fwd-AflII and 111742_rev-SpeI and cloned into the plasmid pRP4-TX [15], putting *diaR1* under the control of the constitutive *tef1* promoter. The plasmids were linearized by digestion with NotI and transformed into *T. reesei* QM6a Δpyr4, selecting for uridine prototrophy on Mandels-Andreotti medium [12] without peptone and 1% (*w*/*v*) glucose. In the resulting strain, OEdiaR1, the *pyr4* locus is reestablished, and the DiaR1 expression cassette is inserted upstream of the *pyr4* promoter (Figure S18).

For the deletion of *dia1-5*, a split marker strategy was used. In brief, the 5′ and the 3′ flanks of the genes were fused to overlapping fragments of the hygromycin marker, *hph,* from pAN7-1 [16], using a splicing by overlap extension (SOE) PCR (Figure S19). The two fusion PCR products were simultaneously transformed into the recipient strain (QM6a Δmus53 or OEdiaR1). The hygromycin resistance gene, *hph*, is assembled by a crossing-over between the overlapping regions (Figure S19), resulting in hygromycin resistance. The gene deletions were confirmed by suitable PCR assays (Figures S20–S25).

### 2.4. Genotyping

Mycelium was harvested and pressed dry between two sheets of filter paper. Approximately 50 mg were lysed in 1 mL CTAB buffer (1.4 M NaCl, 100 mM Tris-HCl pH 8.0, 10 mM EDTA, 2% CTAB, 1% polyvinylpyrrolidone) with 0.37 g small glass beads, 0.25 g medium glass beads and one large glass bead in a 2 mL screw cap reaction tube using a Fast-Prep-24 (MP Biomedicals, Santa Ana, CA, USA) at 6 m s^−1^ for 30 sec. The samples were incubated at 65 °C for 20 min and finally centrifuged at 12,000× *g* for 10 min. The supernatant was transferred to a 2 mL reaction tube, and the DNA was purified by a phenol–chloroform–isoamyl alcohol extraction, followed by a chloroform extraction. The samples were then treated with RNase A (Thermo Fisher Scientific, Waltham, MA, USA) according to the manufacturer’s instructions and the DNA finally precipitated using isopropanol, washed in 1 mL 70% (*v*/*v*) ethanol, and dissolved in 10 mM Tris-HCl pH 8.0 after drying.

All PCR reactions for genotyping were performed with the OneTaq DNA Polymerase or the Q5 DNA Polymerase (both NEB) according to the manufacturer’s instructions.

### 2.5. Transcript Analyses

Mycelium was harvested and pressed dry between two sheets of filter paper, frozen in liquid nitrogen, and stored at −80 °C for up to a week. Approx. 50 mg of mycelium were disrupted in 1 mL RNAzol RT (Sigma-Aldrich) with 0.37 g small glass beads, 0.25 g medium glass beads and one large glass bead in a 2 mL screw cap reaction tube using a Fast-Prep-24 (MP Biomedicals, Irvine, CA, USA) at 6 m s^−1^ for 30 sec. The samples were centrifuged at 12,000× *g* for 10 min; the supernatant was transferred to a 1.5 mL reaction tube and mixed with ethanol 1:1. The RNA was purified using the Direct-zol RNA MiniPrep Kit (Zymo Research, Irvine, CA, USA) according to the manufacturer’s instructions. Notably, this kit contains a DNase treatment step.

For subsequent RT-qPCR analyses, the total RNA was reverse transcribed using the LunaScript RT SuperMix Kit (New England Biolabs, Ipswich, MA, USA) according to the manufacturer’s instructions. The cDNA was diluted 1:50 in ddH2O, and 2 µL was used as a template in a 15 µL reaction using the Luna Universal qPCR Master Mix (New England Biolabs) on a Rotor-Gene Q (Qiagen, Venlo, The Netherlands). Primers were added, and PCR reaction conditions were chosen according to the manufacturer’s instructions. To calculate the relative transcript abundance, we used the Pfaffl method [17] and the *bzp1* and *tpc1* genes as reference genes [18]. The sequences of all used primers are given in Supplemental Material File S8.

### 2.6. Compound Isolation and NMR

The supernatant obtained from 300 mL culture was lyophilized, and the obtained residue was taken up again in 50 mL water. The obtained clear, aqueous solution was extracted several times with ethyl acetate until no further extraction of UV-active compounds was observable via TLC (in total, 300 mL). The collected organic phases were combined, dried over Na_2_SO_4_, filtered, and evaporated under reduced pressure, and the obtained crude residue was analyzed to prove successful extraction using a Nexera UHPLC system (Shimadzu, Kyoto, Japan), equipped with an SPD-M20A PDA detector (Shimadzu) and an LCMS-2020 single quadrupole mass spectrometer (Shimadzu). Chromatographic separation was performed on a XSelect CSH C18 XP column (Waters Corporation, Milford, MA, USA), with an inner diameter of 3.0 mm, a length of 50 mm, and a 130 Å pore size. Buffer systems A (water + 0.1% formic acid) and B (pure acetonitrile) were used at a flow rate of 1.7 mL min^−1^, starting with 5% B for 0.15 min, followed by an increase from 5 to 98% B over a period of 2.05 min and holding at these conditions for 0.30 min.

Preparative separation was performed on a Buchi Pure C850 Flashprep (Büchi Labortechnik AG, Flawil, Switzerland), equipped with a DAD, using a SunFire Prep C18 OBD column (Waters Corporation), with an inner diameter of 30 mm, 100 mm length and 100 Å pore size. Chromatographic separation used Buffer A (water + 0.1% formic acid) and Buffer B (acetonitrile + 0.1% formic acid) employing the following solvent program at a flow rate of 20 mL min^−1^: After injection, 25% B was applied for 2 min, then it was raised to 40% B over a period of 7 min, followed by an isocratic separation at 40% B for another 7 min. Then, over 30 min, the composition was raised to 95% B and again held at these conditions for 5 min. Fractions were collected and analyzed via the Nexera UHPLC system (Shimadzu) to check for the purity of the fractions.

Respective fractions containing diaporthinic acid were evaporated under reduced pressure, dried in high vacuum and taken for NMR measurements at 297 K on an Avance III HD 600 spectrometer (Bruker, Billerica, MA, USA). Obtained spectra were calibrated to the residual solvent peak [19].

### 2.7. Mic Assays

The antibacterial and antifungal MIC assays were performed in 200 µL reactions in a 96-well plate with a flat bottom. First, a serial dilution of diaporthinic acid was prepared in DMSO with the following concentrations: 12.8, 6.4, 3.2, 1.6, 0.8, 0.4, 0.2, 0.1 mg mL^−1^. These solutions were added to double-strength RPMI 2% G medium (RPMI 1640 with L-glutamine without sodium bicarbonate (Thermo Scientific), 20.8 g L^−1^; MOPS, 69.06 g L^−1^; glucose, 36 g L^−1^; pH adjusted to 7.0 with NaOH) for the antifungal assays or Müller–Hinton broth for the bacterial assays. A total of 100 µL of these spiked media was transferred to individual wells, and then 100 µL of the respective bacterial or fungal inocula was added in quadruplicates.

The *E. coli* FDA strain Seattle 1946 (ATCC 25922) and *B. subtilis* strain Marburg (type strain, DSM 10) inocula were prepared by cultivating the bacteria on LB plates (10 g L^−1^ peptone, 5 g/L^−1^ yeast extract, 10 g L^−1^ NaCl) at 37 °C overnight. Several distinct colonies were suspended in Müller–Hinton broth, and the cell density was adjusted to an OD600 of 0.02. The *S. cerevisiae* inoculum was prepared by incubating the strain Sa-07140 (type strain, DSM 70449) on YPD agar (20 g L^−1^ peptone, 20 g L^−1^ glucose, 10 g L^−1^ yeast extract) at 30 °C for 48 h. Several distinct colonies were suspended in sterile, distilled water. The *A. nidulans* inoculum was prepared by cultivating *A. nidulans* FGSC A4 on potato dextrose agar plates at 30 °C for 1 week. Spores were harvested using sterile cotton swabs and resuspended in sterile, distilled water. For *S. cerevisiae* and *A. nidulans*, the cell or spore density was adjusted to an OD530 of 0.1. The bacterial MIC assays were incubated at 37 °C for 24 h, and the antifungal assays at 30 °C for 24 h. Finally, the optical density was measured on a GloMax microplate reader (Promega, Madison, WI, USA) at 600 nm (*E. coli* and *B. subtilis*) or at 560 nm (*S. cerevisiae* and *A. nidulans*).

### 2.8. Hplc-Ms/ms Analysis

LC-MS grade acetonitrile (ACN) and LC-MS grade formic acid were acquired from VWR chemicals (Radnor, PA, USA). Water (H_2_O) was purified in-house using a Barnstead™ Smart2Pure™ Water Purification System from Thermo Fisher Scientific (Waltham, MA, USA). Alternariol (CAS #641-38-3) standard, Catalog #C5061 (Batch No. 1), was purchased from APExBIO (Houston, TX, USA).

The frozen culture media were thawed, vortexed, and 2 mL of each strain and quadruplicate were transferred into fresh Eppendorf tubes. These tubes were spun down for 2 min at 20,000 g at room temperature. From the supernatant, 10 µL was taken out and diluted 1:10 in 2% ACN + 0.1% FA. The injection volume was 1 µL.

For analysis of the NMR-confirmed diaporthinic acid standard, a stock solution of the compound in DMSO was diluted 1:10 in 2% ACN + 0.1% FA. The commercial alternariol standard (1 mg) was dissolved in 1 mL of 50% ACN (with two drops of 30% NH_4_OH) and further diluted 1:100 in 2% ACN + 0.1% FA (c = 10 ng µL^−1^). The injection volume for both standards was 1 µL.

After dilution of the culture media and the reference standards, the samples were measured employing an untargeted metabolomics workflow in positive ionization mode on a Bruker timsTOF Pro equipped with a VIP-HESI source (Bruker Corporation, Billerica, MA, USA). The frontend was a Thermo Fisher Scientific Vanquish H UHPLC with a Waters Acquity BEH C18 column (150 mm × 1 mm ID, 1.7 μm; Waters Corporation, Milford, MA, USA). Solvent A was 0.1% formic acid in water, and solvent B was acetonitrile containing 0.1% formic acid. The following gradient was employed at 40 °C and a constant flow rate of 100 µL min^−1^: 0 min, 2% B; 9 min, 98% B; 12 min, 98% B; 12 min, 2% B, followed by 4 min re-equilibration at 2% B. The timsTOF Pro mass spectrometer (Bruker) was operated without trapped ion mobility spectrometry (TIMS off). For positive ionization mode, source capillary voltage was set to 4500 V and dry gas flow to 8 L min^−1^ at 230 °C. Sheath Gas Flow was set to 4.0 L min^−1^ at a temperature of 200 °C, with active exhaust being activated. Scan mode was set to Auto MS/MS with 12 Hz MS spectra rate and 16 Hz MS/MS spectra rate, resulting in a total cycle time of 0.5 s. The scan range was set from 20 to 800 *m*/*z*.

Individual compounds were quantified on the MS1 level employing the open-source application Skyline version 23.1 [20,21], normalizing to the TIC. Additionally, fragment spectra of the respective compounds were matched to in silico predicted spectra (Table 3 and Figures S26–S35). In silico MS2 fragment prediction and matching were performed using Metfrag 2.6.7. [22].

## 3. Results

### 3.1. Overexpression of Diar1 Leads to Activation of the Dia Bgc

The *dia* BGC in *T. reesei* contains a gene encoding a zinc cluster protein (protein ID 111742 [23], termed DiaR1, see Figure 1 and Table 1). Notably, the predicted protein consists of only 309 amino acids and the NCBI conserved domain search [24,25] only identifies a Gal4-like DNA binding domain (smart00066) at the N-terminus (R12–R52) but no transactivation domain. This is in stark contrast to the standard model for Gal4-like TFs, which entails the presence of both domains in a single protein [26]. Regardless, we decided to overexpress the zinc cluster protein in *T. reesei* by putting the coding region under the control of the constitutive *tef1* promoter and inserting it in front of the *pyr4* gene as described previously [14].

**Figure 2:**
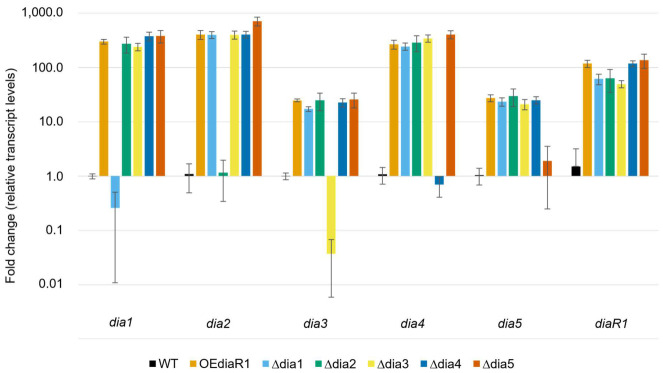
The indicated strains were cultivated in defined medium, and the total RNA was extracted after 48 h. The relative transcript abundances of the indicated genes were measured in the indicated strains via an RT-qPCR analysis and normalized to the transcript levels in the WT strain. The values are the means of biological quadruplicates. The error bars represent standard deviation.

In the resulting strain, *T. reesei* OEdiaR1, we detected elevated transcript levels of all genes within the *dia* BGC compared to the wild-type control strain QM6a Δmus53 (“WT”) (Figure 2, Supplemental Material File S6). The transcript levels of *dia3* and *dia5* were only moderately upregulated (25- and 28-fold, respectively). Importantly, *dia3* and *dia5* were already transcribed in the control strain (Ct values approximately 20 and 22.5, respectively, Supplemental Material File S6), which explains the seemingly modest fold changes in Figure 2. Despite this, their absolute transcript levels in OEdiaR1 were comparable to those of the other *dia* genes (Ct values between 13 and 16, Supplemental Material File S6). For the remaining *dia* genes, no transcripts were detected above background in the control strain (Supplemental Material File S6), indicating that the BGC is silent under the cultivation conditions used.

### 3.2. The Main Compound of the Dia Bgc Is Diaporthinic Acid

Upon successful activation of the *dia* BGC by DiaR1 overexpression, we performed a UV-vis spectrometric screen of the supernatant and compared it to the control strain. We observed several absorption peaks in the spectrum of the OEdiaR1 strain with local maxima at 238 nm, 244 nm, 279 nm, and 330 nm (Figure S1A), indicating the presence of at least one compound that is not present in the control strain. We observed several peaks in the chromatogram during an HPLC-PDA/MS analysis of the supernatant (Figure S1B). Purification of the crude mixture via preparative RP-HPLC allowed the isolation of the substance corresponding to the indicated peak with *m*/*z* (ESI+) = 281 and *m*/*z* (ESI−) = 279. We could identify the compound as diaporthinic acid (**9**) in subsequent 1D- and 2D-NMR experiments (Figure S2).

Next, we performed a minimal inhibitory concentration (MIC) assay to test whether (**9**) possesses antimicrobial properties. In our experimental setup, we could not detect any growth inhibitory effects against *E. coli*, *Bacillus subtilis*, *S. cerevisiae*, or *A. nidulans* in concentrations up to 128 µg mL^−1^ (Figure S3, Supplemental Material File S7).

### 3.3. Dia1, Dia5, and Dia4 Are Essential for Diaporthinic Acid Production

**Figure 3:**
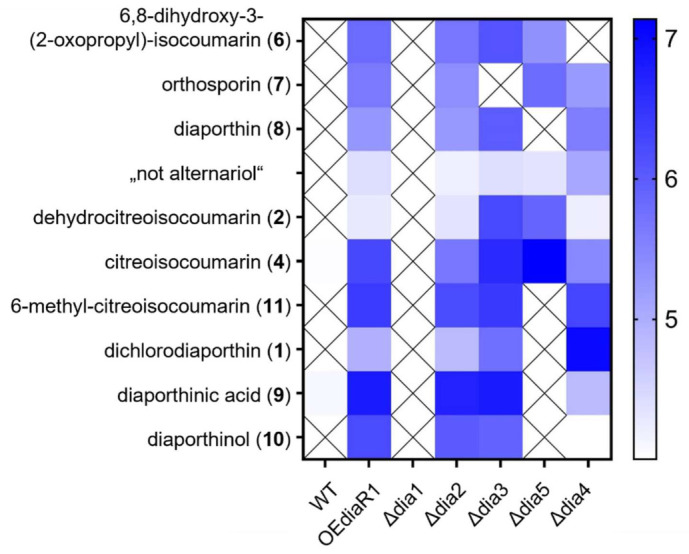
The indicated strains were cultivated in defined medium for 72 h, followed by untargeted metabolomics analysis of their culture supernatants. The heatmap depicts the TIC-normalized and log_10_-transformed peak areas of the detected compounds. X, not detected. The corresponding EICs are shown in Figures S4 and S7–S15.

As mentioned, the *dia* BGC was linked to citreoisocoumarin (**4**), dicholorodiaporthin (**1**) and 8-methyl-dichlorodiaporthin (**3**) in previous studies. Diaporthinic acid (**9**), on the other hand, was only isolated from several fungi, e.g., *P. nalgiovense* (strains IBT 12679, IBT 13296, and IBT 13330) [10] or *Didymella pomorum* (previously *Phoma pomorum*) [11], but has not yet been assigned to a BGC. To test whether (**9**) is in fact originating from the *dia* BGC, we first deleted the *dia1* gene in the OEdiaR1 strain. This gene encodes a PKS, which is the core enzyme of the BGC according to the literature [6]. The resulting strain still exhibited an active BGC, with transcript levels of all genes except the deleted *dia1* remaining comparable to those of the OEdiaR1 strain (Figure 2, Supplemental Material File S6), but we could not detect any of the absorption peaks in the UV-vis spectrum (Figure S1A), nor (**9**) when conducting a HPLC-MS/MS analysis (Figure 3; EIC in Figure S4). This strongly indicates that the activity of the PKS-NRPS Dia1 is essential for the biosynthesis of (**9**), and thus (**9**) originates from the *dia* BGC.

To gain further insight into the biosynthetic pathway of in vivo (**9**), we deleted all of the remaining genes of the *dia* BGC individually in the OEdiaR1 strain. Like in the *dia1* deletion strain, we could still detect high transcript levels of all *dia* genes except the respective deleted genes (Figure 2). In most cases, the transcript levels were similar in these strains to those in the OEdiaR1; only *dia2* was slightly elevated in the Δdia5 strain (Figure 2). However, this might have been an artifact as the reference gene *traff* was lower in these samples than in the other strains (Supplemental Material File S6).

Next, the deletion strains were subjected to untargeted metabolomics measurements by HPLC-MS/MS and compared to the WT and the OEdiaR1 strains. Notably, a commercial NMR verified standard was only available for (**5**), while (**9**) was isolated and verified in-house by NMR as described above. Further, we could also isolate and verify (**1**) and (**4**) by NMR (Figures S5 and S6). The measured NMR spectra fit previously published data [10,27]. Identification of the other compounds was based on exact mass as well as shared MS/MS fragments with (**1**), (**4**) and (**9**). The EICs of all compounds are shown in Figures S4 and S7–S15. Moreover, matching experimental fragmentation spectra to in silico fragmentation predictions of the putative structures revealed high to very high matches. However, as there are no authentic standards commercially available and the amounts were insufficient for NMR analysis, these structures should be treated as putatively belonging to the diaporthin compound class when interpreting the results. The biosynthetic pathway up to (**1**) (Figure 1B), including shunt products, was studied in detail previously [5,6] and is not the focus of this study. Please refer to Table 3 for a list of all detected compounds used for data interpretation in this study.

Matching the transcript levels (Figure 2), we could not detect any *dia* BGC-related compounds in the WT and Δdia1 strain (Figure 3). In the OEdiaR1 strain, we measured high levels of compound (**9**), consistent with the initial HPLC-PDA/MS analysis of the supernatant (Figure S1B). In addition, substantial amounts of compound (**4**) and compounds with masses corresponding to (**10**) and (**11**) were detected (Figure 3), along with weaker signals of compounds matching the masses of (**6**)–(**8**). For (**1**) and compounds with masses matching (**2**) and (**5**), only low levels were observed (Figure 3). Notably, we did not detect (**3**) in any of the tested strains.

Importantly, the compound with an exact mass (observed *m*/*z* = 259.061; theoretical [M + H]^+^ = 259.060) matching (**5**) showed a significant mismatch in retention time and fragmentation pattern compared to the commercially available alternariol standard. Therefore, the detected compound is not (**5**), and we designated it as “not alternariol.” Due to its low abundance, isolation for structural elucidation was not possible. Nonetheless, the compound can still be attributed to the *dia* BGC, as it was not detected in the Δdia1 strain (Figure 3).

The deletion of *dia5* (homolog of *A. oryzae aoiQ*) resulted in an abolishment of compound (**1**) production (Figure 3). In this strain, we could not detect signals for (**8**), (**9**), (**10**), or (**11**) but observed an elevated accumulation of (**4**) and a compound with a mass matching (**2**) compared to the OEdiaR1 strain. The deletion of *dia4* drastically reduced the abundance of (**9**), as well as of a compound with a matching mass for (**10**), and led to a strong accumulation of (**1**), suggesting that (**1**) might be the substrate for Dia4. In the Δdia4 strain, we also observed a slightly stronger signal for “not alternariol” (Figure 3).

### 3.4. Dia2 and Dia3, Essential for Dichlorodiaporthin Formation In Vitro, Are Dispensable In Vivo

Previously, Liu et al. demonstrated that *A. oryzae* DiaA and DiaB work together to synthesize (**2**) and that DiaC and AioQ act simultaneously on (**2**), yielding (**3**) [6]. As mentioned, (**3**) differs from (**1**) by a methyl group that is uniquely added by the *A. oryzae* AoiQ but not by any of its homologs from other fungi [6]. Therefore, (**3**) in *A. oryzae* equals (**1**) in the *T. reesei* biosynthetic pathway. Our results suggest that neither Dia2 nor Dia3 is essential for the biosynthesis of (**1**) in vivo. The deletion of *dia2* did not influence the signal intensities for the compounds with masses matching all anticipated metabolites in comparison to the OEdiaR1 strain (Figure 3). The deletion of *dia3* did not result in an abolishment of (**1**) or (**9**) production in *T. reesei*, or any other intermediate other than (**7**) (Figure 3). We even observed stronger signals for (**1**) and the assumed (**2**), (**6**), and (**8**) in the Δdia3 strain compared to the OEdiaR1 strain (Figure 3).

At this point, we want to mention that the accumulated biomass of the Δdia3 strain is strongly reduced in comparison to the other strains (Figure S16). As mentioned above, *dia3* is also expressed in the wild-type strain (Supplemental Material File S6), indicating that Dia3 is not exclusively participating in the biosynthetic pathway of the *dia* BGC, but might also catalyze other reactions. Thus, we sought to determine whether the reduced growth of Δdia3 is specifically due to the absence of Dia3 in the biosynthetic *dia* pathway or a consequence of its overall loss. Therefore, we deleted the *dia3* gene in the wild-type strain. The resulting strain WT/Δdia3 has no growth defects and grows similarly to the wild-type strain (Figure S16). This strongly indicates that Dia3 plays an important role during the biosynthesis of (**1**) and (**9**), but this is not necessarily exclusively its previously reported catalytic activity.

## 4. Discussion

In this study, we successfully activated the *dia* BGC in *T. reesei* by overexpressing *diaR1* (protein ID 111742), encoding a protein containing only a DNA-binding domain, and found diaporthinic acid (**9**) to be the main product of the BGC. As mentioned, (**9**) was first identified together with (**1**) and (**10**) in three isolates of *P. nalgiovense* (IBT 12679, IBT 13296, and IBT 13330) [10] and was also detected together with (**1**), (**4**), (**8**), (**10**), and (**11**) in 27 of 27 tested *D. pomorum* strains [11]. The genomes of the *P. nalgiovense* and *D. pomorum* strains used in the previous studies are not available, but we found homologs of the *dia* BGCs in the genomes of sequenced strains (Figure 1 and Table S1). Taken together, we postulate that the *dia* BGC is responsible for the production of (**9**) not only in *T. reesei* but also in *P. nalgionvense*, *D. pomorum*, and possibly other fungi that contain this BGC [6].

We observed that the main product of the *dia* pathway in *T. reesei* is (**9**), which accumulates in extraordinary abundance in the OEdiaR1 strain in relation to the other metabolites. The deletion of *dia5* (homolog of *aoiQ*) resulted in a complete abolishment of the halogenated (**1**), as well as all 6-O methylated compounds (**8**), (**9**), (**10**), and (**11**). This is in accordance with Liu et al. and the bifunctional nature of Dia5 and AoiQ as methyltransferases and halogenases. In contrast to AoiQ, however, Dia5’s methyltransferase activity is selective for the 6-hydroxy group and does not conduct methylation of the 8-hydroxy group [28], thus (**3**) in *A. oryzae* corresponds to (**1**) in *T. reesei*. The absence of (**9**) and the compounds with the masses of (**10**) and (**11**) also corroborates that Dia5 acts as the exclusive methyltransferase in the biosynthetic pathway.

**Figure 4:**
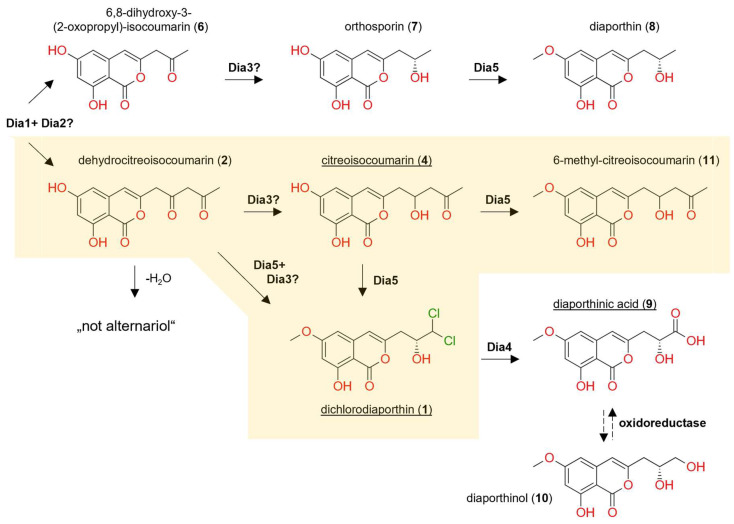
Proposed biosynthetic pathways towards diaporthin (**8**), diaporthinic acid (**9**), and diaporthinol (**10**), all originating from the *dia* BGC in *T. reesei*. Compounds determined by NMR in this study are underlined. The rest of the compounds were attributed by matching masses and high matching values of detected and in silico predicted MS2 fragments (Table 3). The biosynthetic pathway suggested for *A. oryzae* by Liu et al. [6] is indicated by the yellow background (refer also to Figure 1B). Please note that (**11**) corresponds to 6-methyl-citreoisocoumarin and (**1**) to (**3**) in Figure 1B due to the unique ability of *A. oryzae* AoiQ to catalyze the O-8 methylation in addition to the O-6 methylation [6]. Dashed arrows indicate a supposed interconversion reaction between (**9**) to (**10**).

In the Δdia4 strain, we detected substantially lower amounts of (**9**) compared to OEdiaR1, and a strong accumulation of (**1**), which suggests that (**1**) is the substrate for Dia4. Dia4 contains a p-cresol methylhydroxylase (PCMH)-type FAD-binding domain (Uniprot ID G0RVA9). This domain is found in various oxidases and dehydrogenases according to its Interpro entry (IPR016166). Dia4 is therefore a suitable candidate for the oxidation of (**1**), yielding (**9**) in *T. reesei*. The proposed biosynthetic pathways based on our results and previous results from the *A. oryzae dia* BGC [6] are compiled in Figure 4.

Further, we speculate that the synthesis of the assumed (**10**) might be the product of a reduction of (**9**), as (**10**) arises only downstream of Dia5 and Dia4, and the relation of the abundances of (**9**) and (**10**) remains similar in all strains (Figure 3). This reduction might be catalyzed by an unspecific oxidoreductase present in the proteome of *T. reesei*. At this point, this is just speculation; the possible reaction is therefore included in Figure 4 with dashed arrows.

In this study, we could attribute further metabolites to the *T. reesei dia* BGC, i.e., compounds with the same exact masses as (**5**), (**7**) and (**8**). Comparison to a commercial standard revealed that the presumed detected compound is actually not (**5**), despite exhibiting the same exact mass. “Not alternariol” shows a mass difference of 18.01 Da compared to (**2**), which suggests that “not alternariol” might be a dehydration (-H_2_O) derivative of (**2**).

Compounds (**7**) and (**8**) were previously reported to be the products of the *aoi* BGC in *A. oryzae* [5,6]. In this study, we could attribute compounds with the respective matching masses to the *dia* BGC (Figure 3). In the Δdia5 strain, we observed a complete abolishment of the assumed (**8**), matching the results from Liu et al. [6]. There, AoiQ was shown to be responsible for the methylation of (**7**), yielding (**8**). If the compounds with masses matching (**6**)–(**8**) are indeed the presumed compounds, this may indicate that *T. reesei* Dia1 is able to release both (**2**) and (**6**), in contrast to *A. oryzae* DiaA, which was reported to only release (**2**) [6]. The suggested pathway is represented in Figure 4. Importantly, this proposed pathway would also be consistent with previous reports of co-isolation of diaporthin-related metabolites (**1**), (**4**), (**8**), (**9**), (**10**) and (**11**) in *D. pomorum* [11].

DiaB, the Dia2 homolog in *A. oryzae*, was previously speculated to compensate for the missing enoyl-reductase in DiaA and to catalyze the release of (**6**) [7]. Dia1 shares the same domain composition as DiaA, likewise lacking an enoyl reductase domain. Interestingly, we detected the same metabolites in the Δdia2 strain as in the OEdiaR1 strain (Figure 3). As the release of (**2**) (and possibly also (**6**)) from Dia1 is highly unlikely to occur spontaneously, it is plausible that another enoyl reductase complements Dia2. A BLAST analysis, (https://blast.ncbi.nlm.nih.gov/Blast.cgi accessed on 27 August 2024) [29] against the predicted *T. reesei* proteome using the *A. oryzae* DiaB protein sequence as query on the JGI genome portal returns an alternative for Dia2 (protein ID 60,671 [23], Figure S17A), a protein containing an uncharacterized subgroup of MBL-fold metallo hydrolase domain (cd07722) and a beta-lactamase associated winged helix domain (pfam17778).

Analogously, the SDR Dia3 was not essential for the biosynthesis of (**1**) and (**9**) (Figure 3), which is in stark contrast to the in vitro observations by Liu et al. [6]. Deletion of *dia3* in the OEdiaR1 strain did not halt the biosynthetic pathway, but it altered the relative abundances of several intermediates. Compounds matching the masses of (**2**) and (**6**) accumulated in the absence of Dia3 (Figure 3), consistent with the proposed pathway, as (**2**) and (**6**) are assumed substrates of Dia3 (Figure 4). Also in line with the pathway, no signals for (**7**) were detected (Figure 3), which is the proposed reduction product of (**6**) by Dia3 (Figure 4). Surprisingly, we still detected a compound matching the mass of (**8**), which is downstream of (**7**) (Figure 4). Equally unexpected, we did not observe lower abundances of (**1**) and (**4**) (Figure 3), both assumed products of Dia3 (Figure 4).

For DiaC, a BLAST analysis [28] using the *A. oryzae* DiaC as query returned several hits (Figure S17B); the top five are all SDRs, like DiaC and Dia3. One or more of them might also accept (**2**) (and possibly (**6**)) as substrate and catalyze the necessary reduction reaction in the absence of Dia3. If this alternative reductase reduces (**6**) to (**7**) at a lower rate than Dia3, the absence of detectable (**7**) could be explained. In such a scenario, Dia5 may methylate the small amounts of (**7**) to yield (**8**) more rapidly than (**6**) can be reduced to (**7**).

Although the role of Dia3 in the biosynthetic pathway appears to have been compensated, we observed a strong reduction in biomass (Figure S16). This may indicate that Dia3 is required for self-resistance, or that its absence leads to the accumulation of self-toxic intermediates or derivatives due to the action of alternative reductases or other enzymes.

However, Dia3 (protein ID 123,964 [23]) was previously described as being associated with cellulase signal transduction (Genebank CF653652) [29]. In that study, *dia3* transcripts were detected in elevated amounts shortly after a sophorose pulse, as well as during cultivation on glucose, and to some extent also on glycerol, but not on cellulose directly [12]. These expression patterns suggest that Dia3 is not directly linked to cellulose degradation but may be involved in other processes. Importantly, we detected relatively high basal transcript levels of *dia3* in the wild-type strain (Figure 2, Supplemental Material File S6). Taken together with the presumed compensation of Dia3 activity in the proposed pathways to (**8**) and (**9**) (Figure 3 and Figure 4), this further supports the idea that Dia3 and other SDRs may serve additional biological roles in *T. reesei* and are likely able to compensate for each other.

## Figures and Tables

**Table 1:** Genes in the *dia* BGC (see also Supplemental Files S1–S4 for the corresponding GenBank files).

Gene Name	Protein ID	Enzyme Class	Homolog in *A. oryzae*	Homolog in *A. nidulans*	Homolog in *P. nalgiovense*
*dia1*	81964	polyketide synthase	*diaA*	*pkgA*	*ngvA*
*dia2*	69625	beta-lactamase-like	*diaB*	*pkgB*	*ngvB*
*dia3*	123964	dehydrogenase	*diaC*	pkgC	*ngvC*
*dia5*	69652	bifunctional flavin-dependent halogenase/methyltransferase	*aoiq*	*aoiq*	*ngvE* (FDH) *ngvF* (MT)
*dia4*	69650	FAD-dependent oxidoreductase	*diaD*	pkgD	*ngvD*
*diaR1*	111742	zinc cluster protein	XM_003190268.1	ANIA_07073	n/a

**Table 2:** *T. reesei* strains used in this study.

Strain Name	Genotype	Source
QM6a Δmus53 (“WT”)	Δ*mus53*::*bar*	Steiger et al. [13]
QM6a Δpyr4	Δ*mus53*::*bar* Δ*pyr4*	Derntl and Kiesenhofer et al. [14]
OEdiaR1	Δ*mus53*::*bar* Ptef::*diaR1* (*pyr4*)	This study
Δdia1	Δ*mus53*::*bar* Ptef::*diaR1* (*pyr4*) Δ*dia1*::*hph*	This study
Δdia2	Δ*mus53*::*bar* Ptef::*diaR1* (*pyr4*) Δ*dia2*::*hph*	This study
Δdia3	Δ*mus53*::*bar* Ptef::*diaR1* (*pyr4*) Δ*dia3*::*hph*	This study
Δdia5	Δ*mus53*::*bar* Ptef::*diaR1* (*pyr4*) Δ*dia5*::*hph*	This study
Δdia4	Δ*mus53*::*bar* Ptef::*diaR1* (*pyr4*) Δ*dia4*::*hph*	This study
WTΔdia3	Δ*mus53*::*bar* Δ*dia3*::*hph*	This study

**Table 3:** Analytes, including sum formula and exact mass information.

Assumed Compound	Sum Formular	Exact Mass [Da]	Matching to NMR Confirmed Standard?	Metfrag #Frag Matched	Metfrag Explained IntCov. [%]
dichlorodiaporthin (**1**)	C_13_H_12_Cl_2_O_5_	318.006 *	Indicated by ^1^H	21	82.02
dehydrocitreoisocoumarin (**2**)	C_14_H_12_O_6_	276.063	n/a	22	81.56
citreoisocoumarin (**4**)	C_14_H_14_O_6_	278.079	Confirmed by ^1^H and ^13^C	15	83.19
alternariol (**5**)	C_14_H_10_O_5_	258.053	NO	3	23.08
6,8-dihydroxy-3-(2-oxopropyl)-isocoumarin (**6**)	C_12_H_10_O_5_	234.053	n/a	22	77.34
orthosporin (**7**)	C_12_H_12_O_5_	236.069	n/a	17	92.03
diaporthin (**8**)	C_13_H_14_O_5_	250.084	n/a	18	89.69
diaporthinic acid (**9**)	C_13_H_12_O_7_	280.058	Confirmed by ^1^H and ^13^C	9	96.63
diaporthinol (**10**)	C_13_H_14_O_6_	266.079	n/a	30	68.73
6-methyl-citreoisocoumarin (**11**)	C_15_H_16_O_6_	292.095	n/a	11	75.30

* presence of characteristic isotopic pattern for a dichloro-species. n/a: no standards were available.

## Data Availability

The metabolomics datasets generated during and/or analyzed during the current study are available in the TU Wien Research Data Repository, https://doi.org/10.48436/w9j5t-wz734.

## Supplementary Materials

The following supporting information can be downloaded at: https://www.mdpi.com/article/10.3390/jof12060402/s1.
