# Supplementary material for: In Vivo Characterization of the dia Biosynthetic Gene Cluster Reveals Diaporthinic Acid as Its Main Product
Source: J Fungi (Basel). 2026 Jun 1;12(6):402. doi: 10.3390/jof12060402 (PMC13301986; doi:10.3390/jof12060402)
Supplement: Supplementary file 1 [file jof-12-00402-s001.zip › Supplemental_File_5_Figures_and_Tables.pdf]

In vivo activation of the dia BGC allows consolidation of the biosynthetic pathways of diaporthin, dichlorodiaporthin, diaporthinic acid, and diaporthinol

Isabella Burger[1,2‡], Simon Leonhartsberger[3‡], Kathrin Peikert[4], Lukas Fourtis[4], Polina Atanasova[4], Lara T.S. Kramer[4], Richard Fried[3], Christian Stanetty[3], Florian Rudroff[3], Ruth Birner-Gruenberger[1], Robert L. Mach[4], Astrid R. Mach-Aigner[4], Matthias Schittmayer[1]*, Christian Zimmermann[4]*

[1] Institute of Chemical Technologies and Analytics, TU Wien, Vienna, 1060, Austria

[2] Department of Analytical Chemistry, Faculty of Chemistry, University of Vienna, Vienna, 1090, Austria

[3] Institute of Applied Synthetic Chemistry, TU Wien, Vienna, 1060, Austria

[4] Institute of Chemical, Environmental and Bioscience Engineering, TU Wien, Vienna, 1060, Austria

‡ IB and SL contributed equally to this study

*Email: matthias.schittmayer@tuwien.ac.at

*Email: christian.zimmermann@tuwien.ac.at

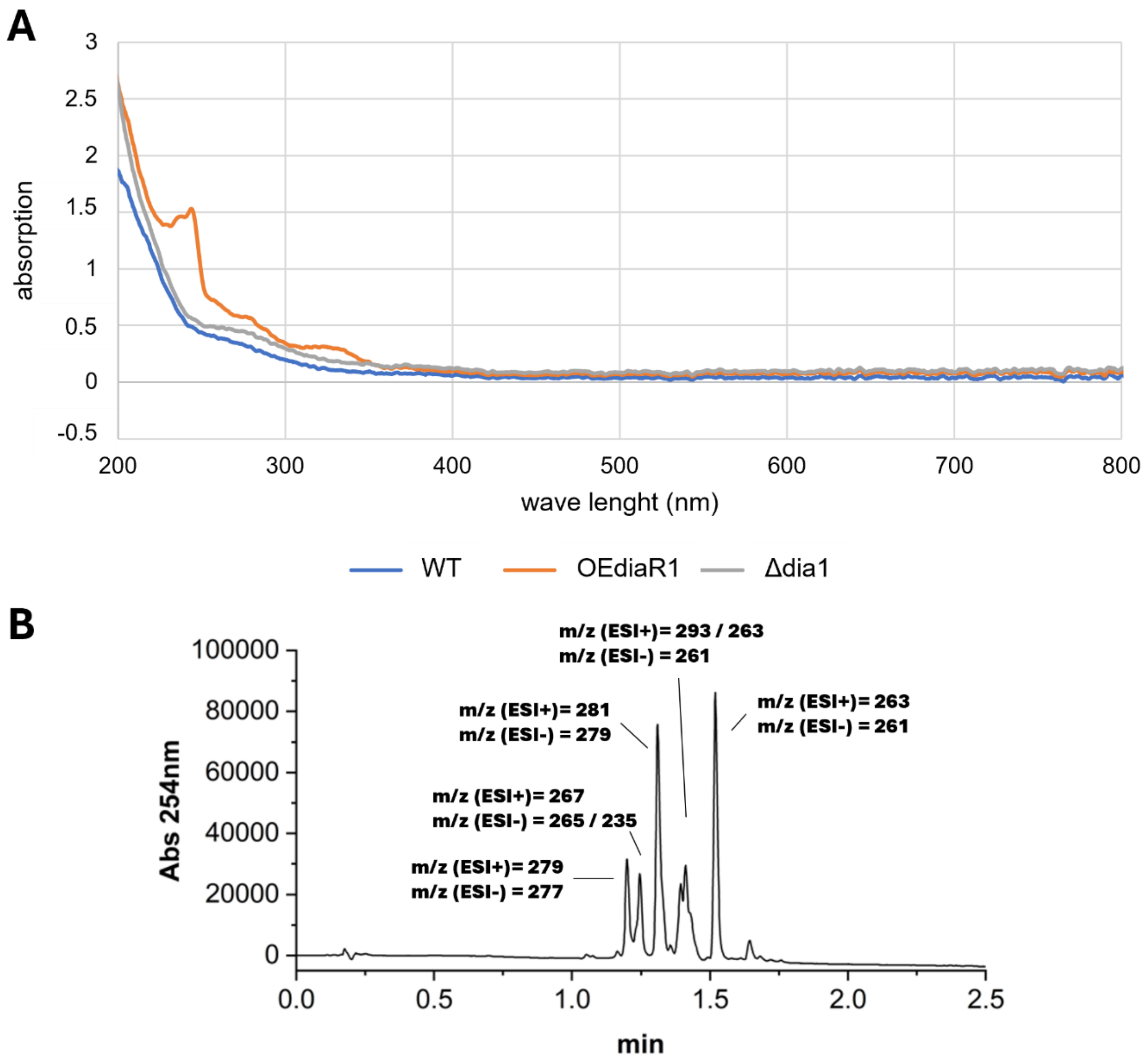

Figure S1. (**A**) The *T. reesei* strains QM6a Δmus53 (WT), OEdiaR1, and Δdia1 were cultivated in MAM + glycerol for 72 hours and the absorption spectrum of the resulting supernatant measured. (**B**) The supernatant of OEdiaR1 was subjected to a HPLC-PDA/MS analysis.

A

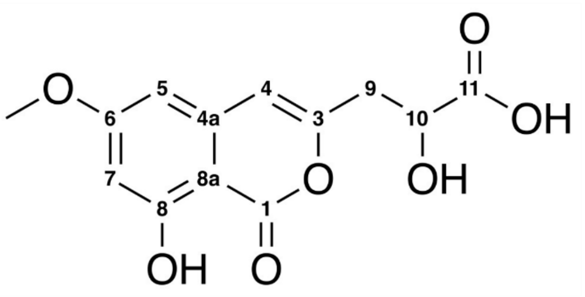

B

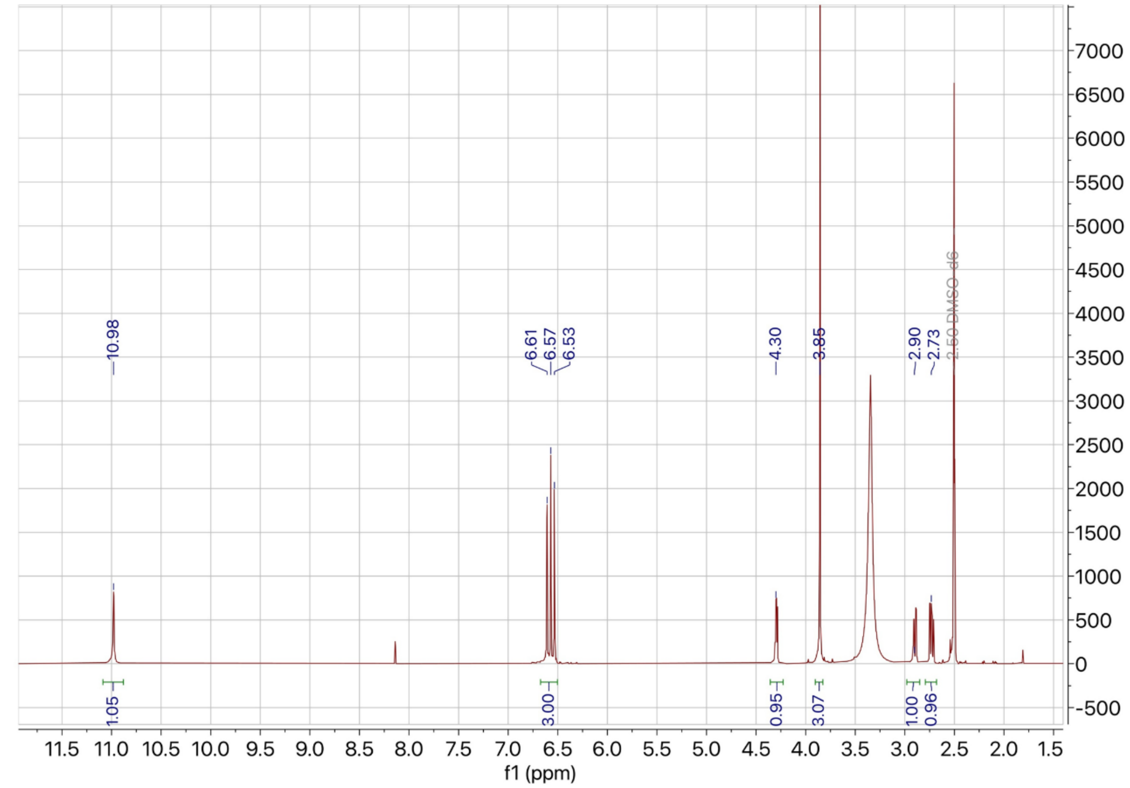

Figure S2. NMR spectra of the peak with *m/z* (ESI+) = 281 and *m/z* (ESI-) = 279 in Fig. S1B.

(**A**) structure formular of diaporthinic acid (**9**)

(**B**) 1H-NMR (600MHz, d6-DMSO): δ = 2.73 (dd, J = 8.6, 14.7 Hz, 1H, H9-A), 2.90 (dd, J = 4.4, 14.7 Hz, 1H, H9-B), 3.85 (s, 3H, OCH3), 4.30 (dd, J = 4.4, 8.6 Hz, 1H, H10), 6.53 (d, J = 2.3 Hz, 1H, H7), 6.57 (s, 1H, H4), 6.61 (d, J = 2.3 Hz, 1H, H5), 10.98 (s, 1H, COOH) ppm

(**C**) 13C-NMR (600MHz, d6-DMSO): δ = 37.86 (C9), 55.96 (OCH3), 67.60 (C10), 99.38 (C8a), 100.52 (C7), 101.30 (C5), 106.05 (C4), 139.39 (C4a), 154.11 (C3), 162.52 (C8), 165.29 (C1), 166.52 (C6), 174.40 (C11) ppm

(**D-F**) Additional 2D-NMR spectra used for structure elucidation: COSY, HSQC, HMBC.

Figure S2. (continued)

C

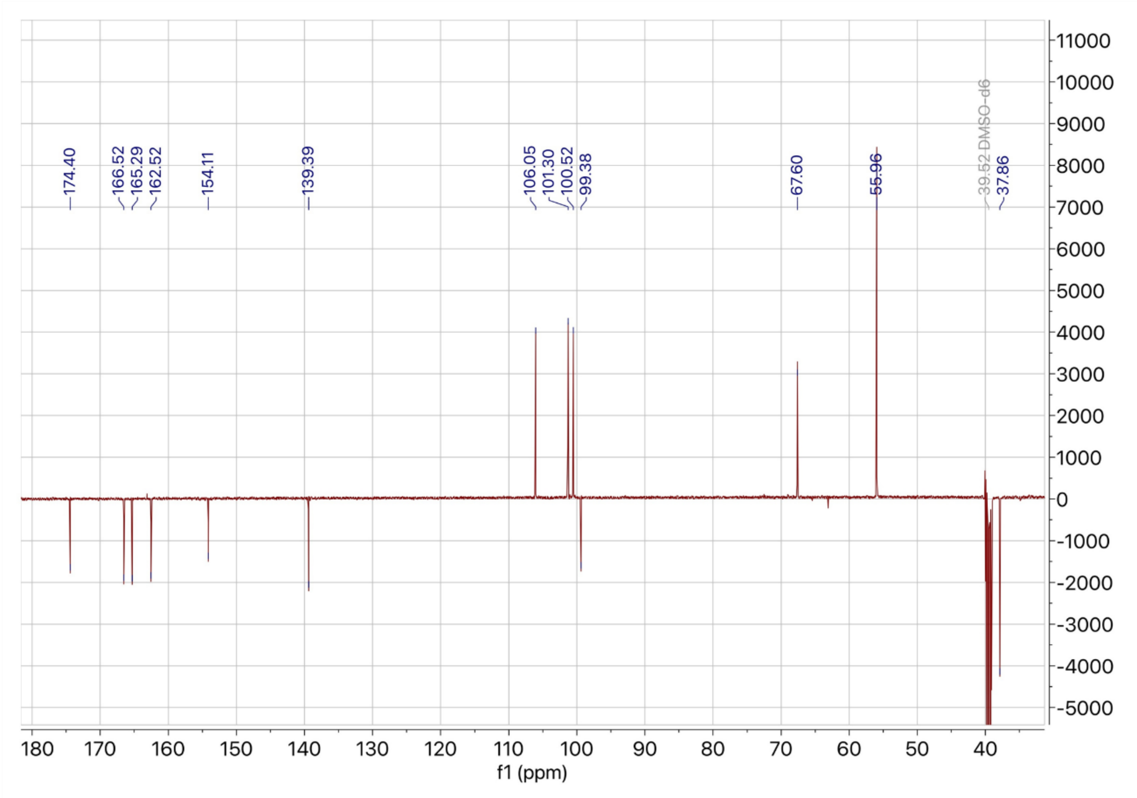

D

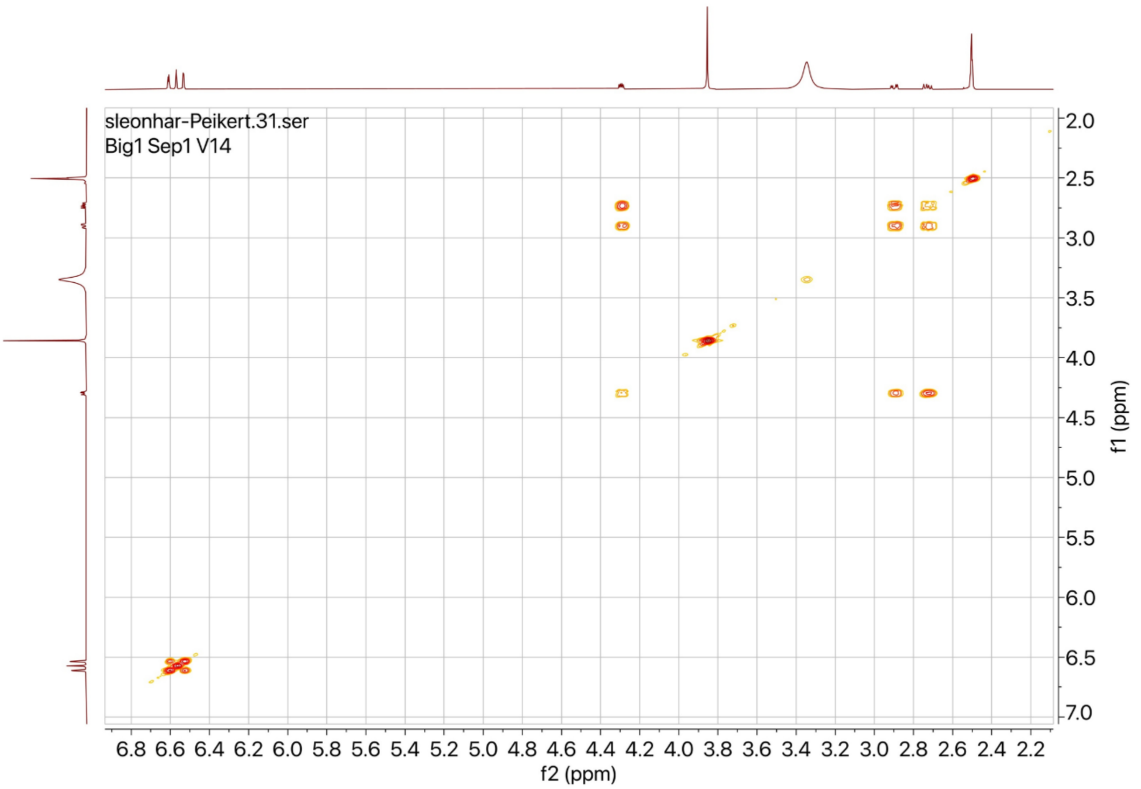

Figure S2. (continued)

E

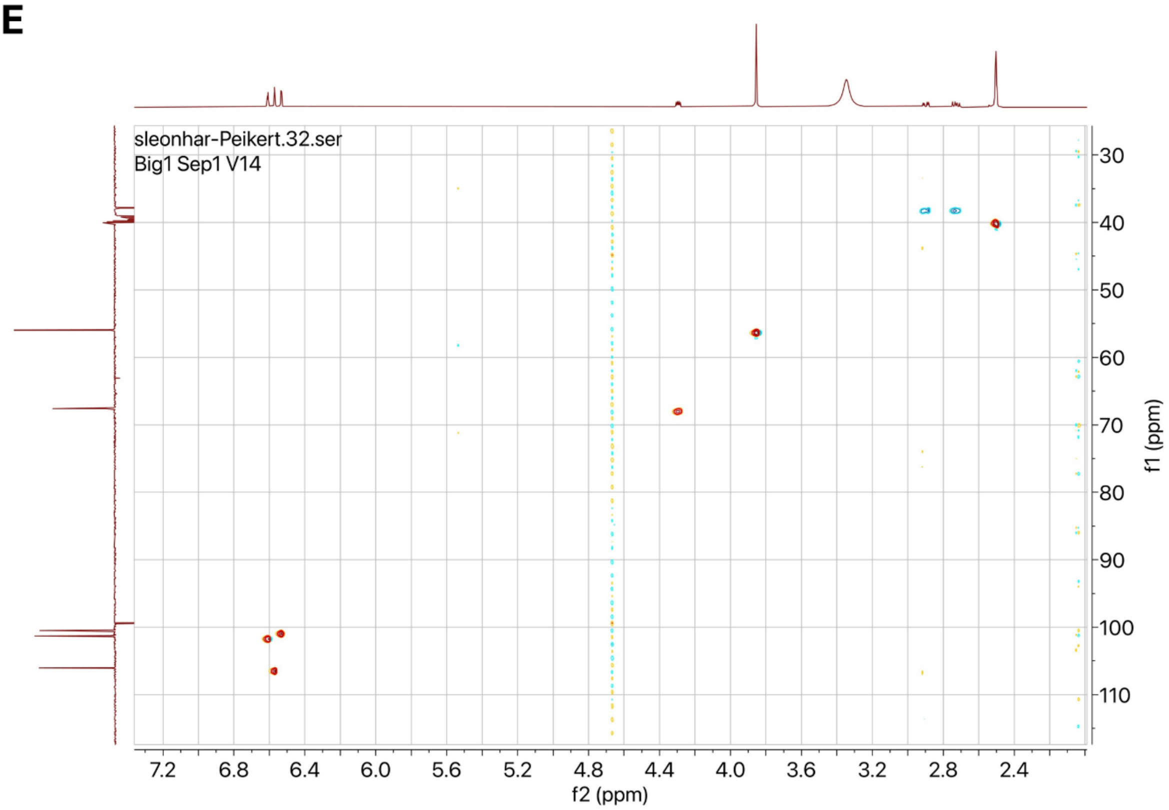

F

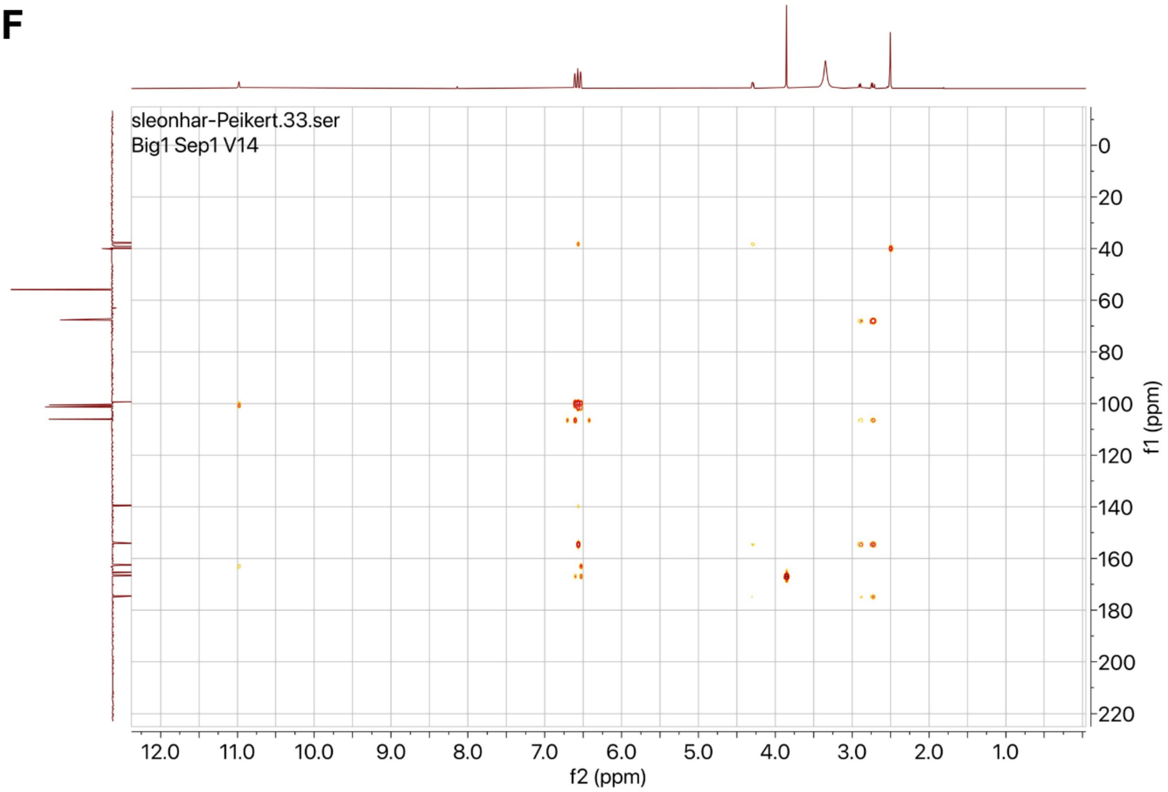

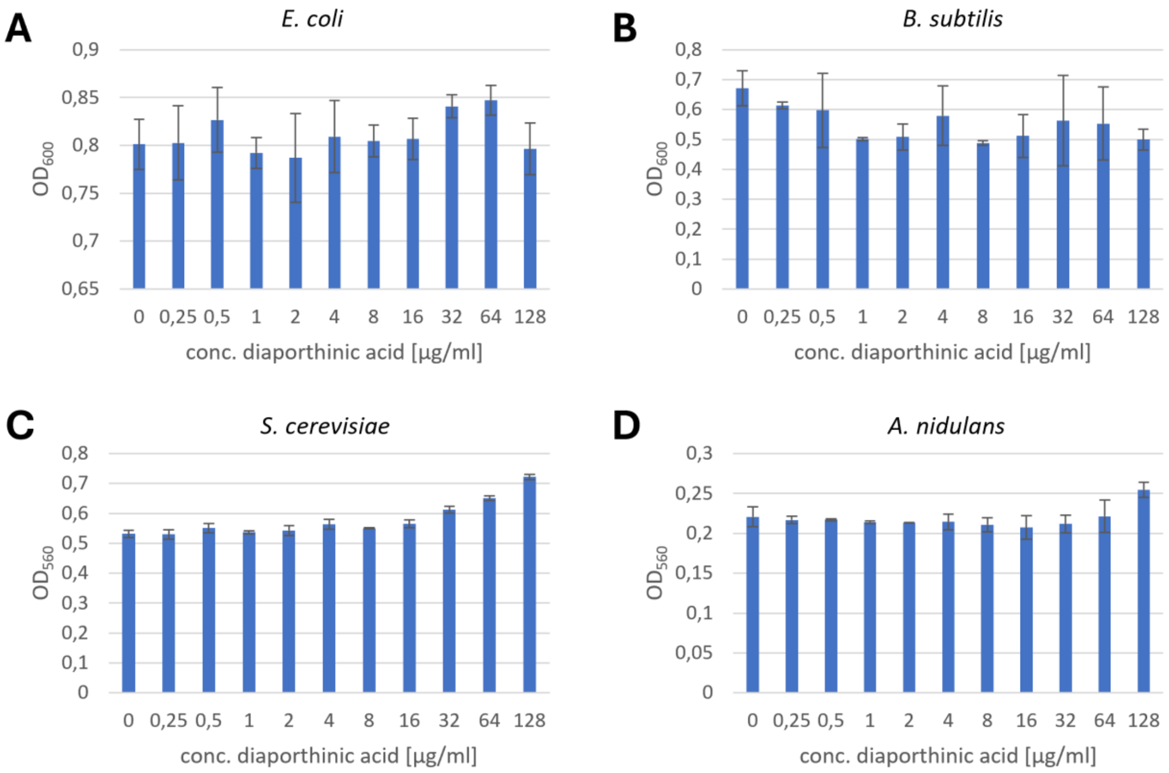

Figure S3. The indicated microorganisms were tested from sensitivity against diaporthinic acid in a MIC assay. The obtained culture density is shown in dependency of the diaporthinic acid concentration.

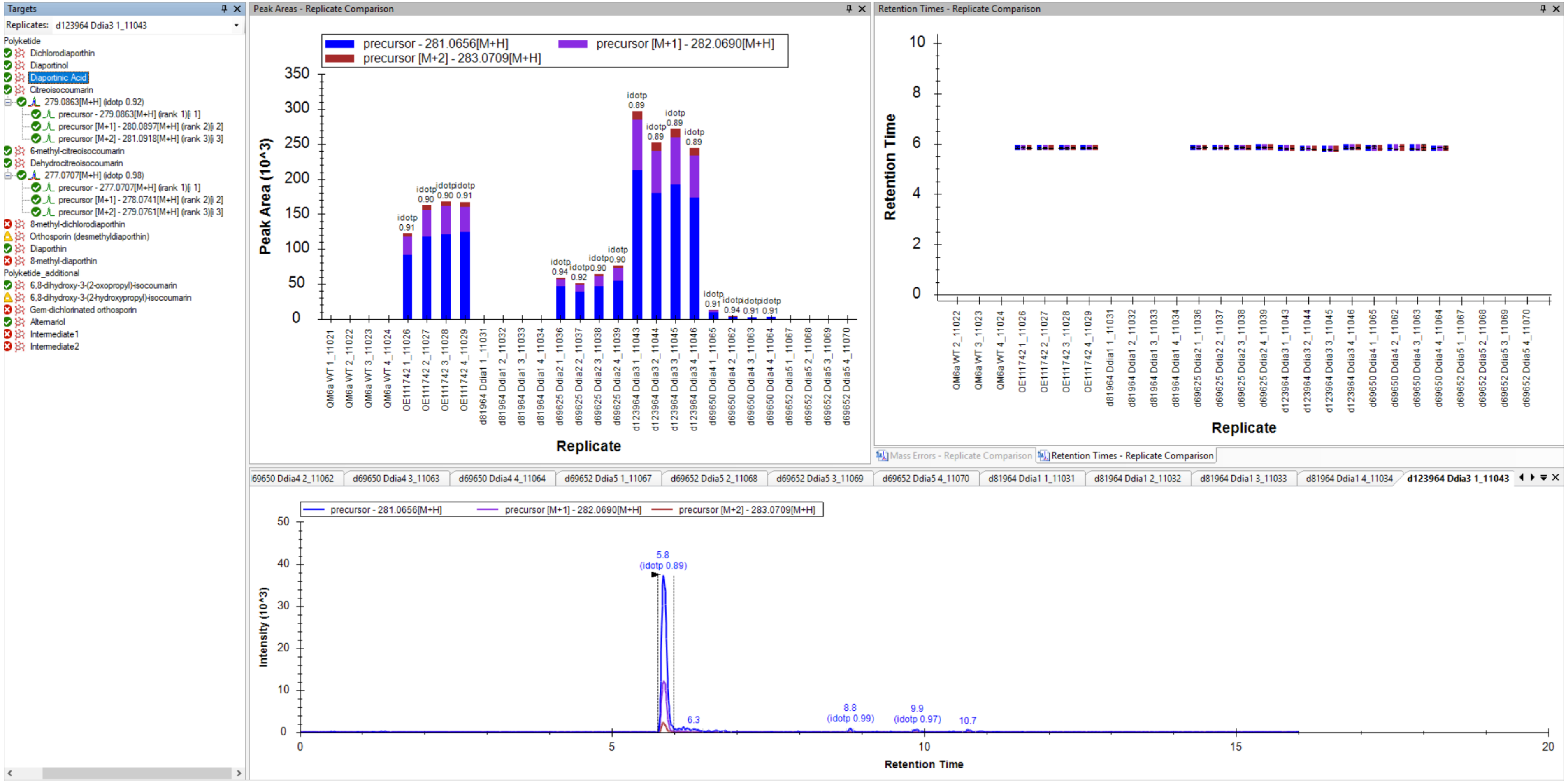

Figure S4. EIC of diaporthinic acid (**9**).

A

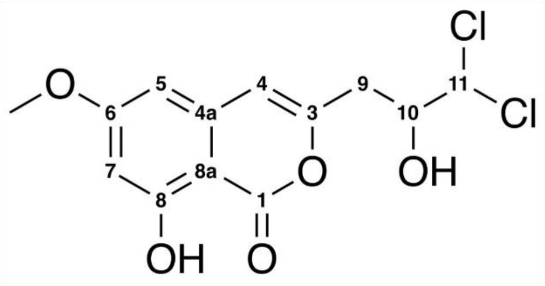

B

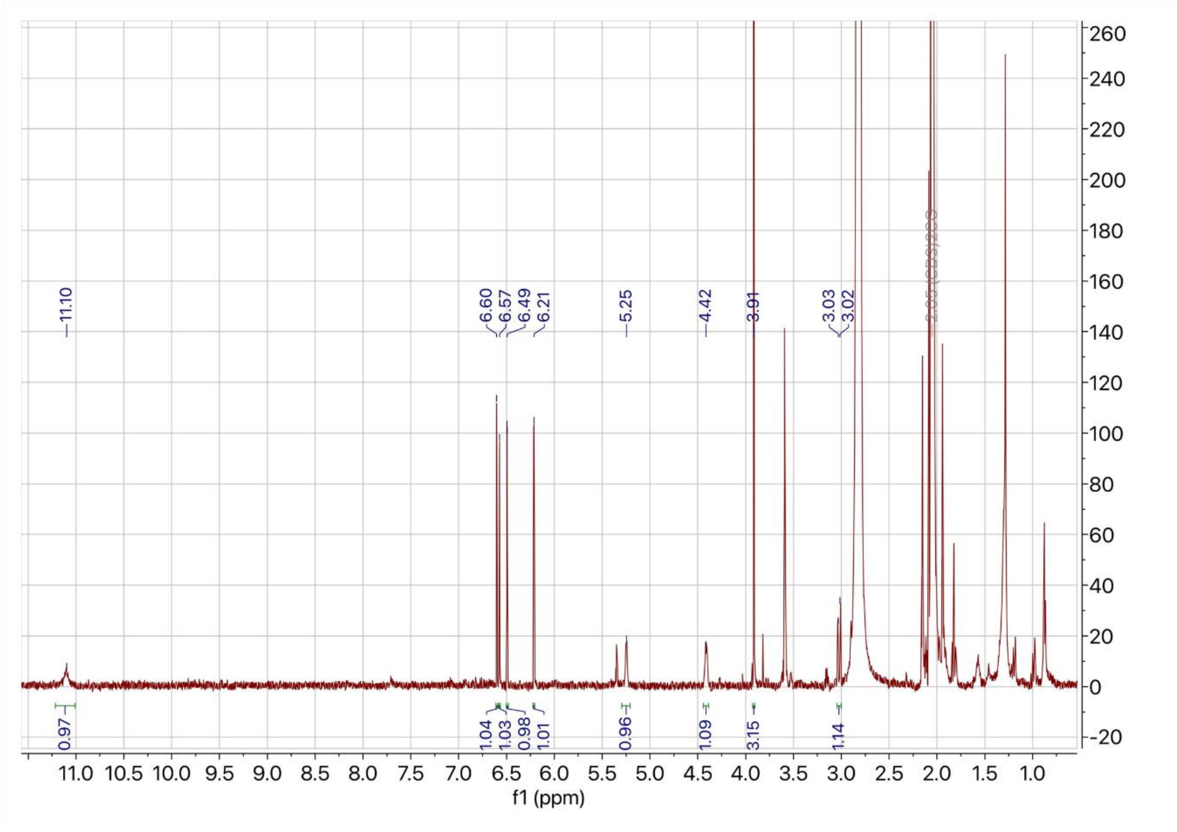

Figure S5. NMR spectrum of dichlorodiaporthin (1).

(**A**) structure formular of dichlorodiaporthin (1)

(**B**) 1H-NMR (400MHz, d6-acetone): δ = 3.02 (dd, J = 14.57, 3.52 Hz, 1H), 3.91 (s, 3H), 4.42 (m, 1H), 5.25 (s, 1H), 6.21 (d, J = 3.37 Hz, 1H), 6.49 (d, J = 2.31 Hz, 1H), 6.57 (d, J = 2.31, 1H), 6.60 (s, 1H), 11.10 (s, 1H) ppm
(assumed signal (dd) at 2.80ppm overlaps with strong water peak)

A

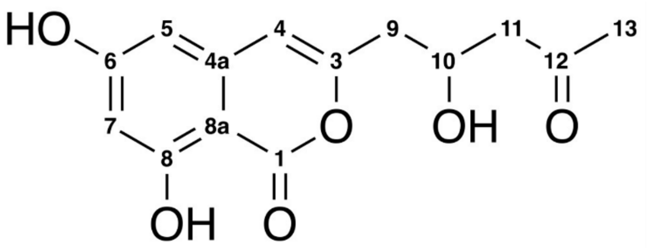

B

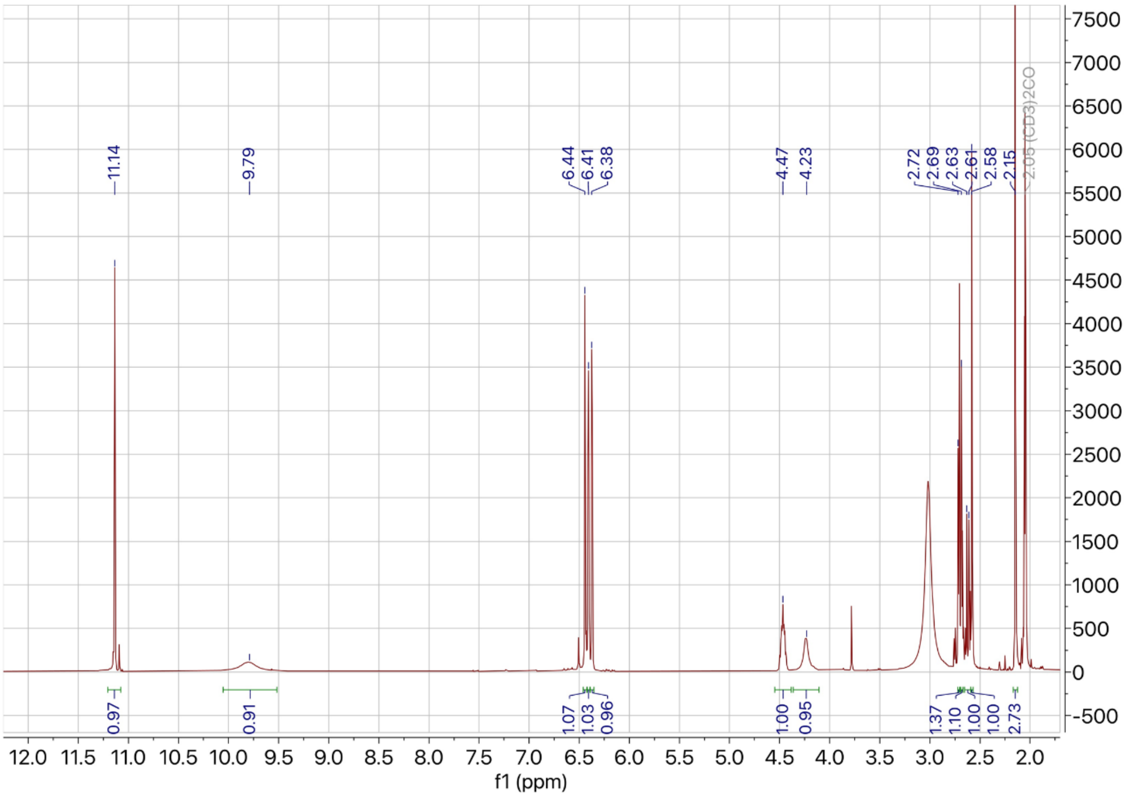

Figure S6. NMR spectra of citreoisocoumarin (4).

(**A**) structure formular of citreoisocoumarin (4).

(**B**) 1H-NMR (400MHz, d6-acetone): δ = 2.15 (s, 3H), 2.58 (m, 1H), 2.62 (m, 1H), 2.69 (d, J = 4.84 Hz, 1H), 2.72 (d, J = 4.84 Hz, 1H), 4.23 (m, 1H), 4.47 (m, 1H), 6.38 (d, J = 1.91 Hz, 1H), 6.41 (d, J = 1.91 Hz, 1H), 6.44 (s, 1H), 9.79 (s, 1H), 11.14 (s, 1H) ppm

(**C**) 13C-NMR (400MHz, d6-acetone): δ = 30.65, 41.83, 50.70, 66.09, 99.89, 102.30, 103.40, 106.67, 140.81, 155.79, 164.51, 166.38, 167.06, 206.33 ppm

Figure S6. (continued)

C

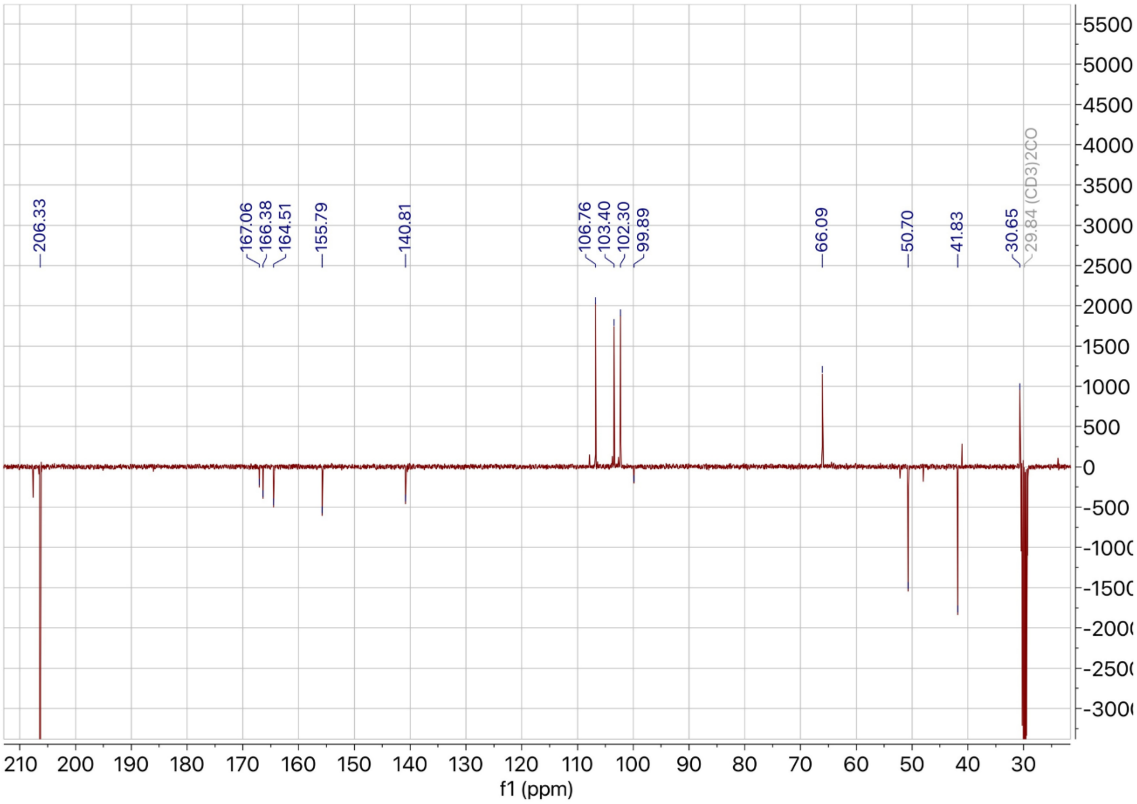

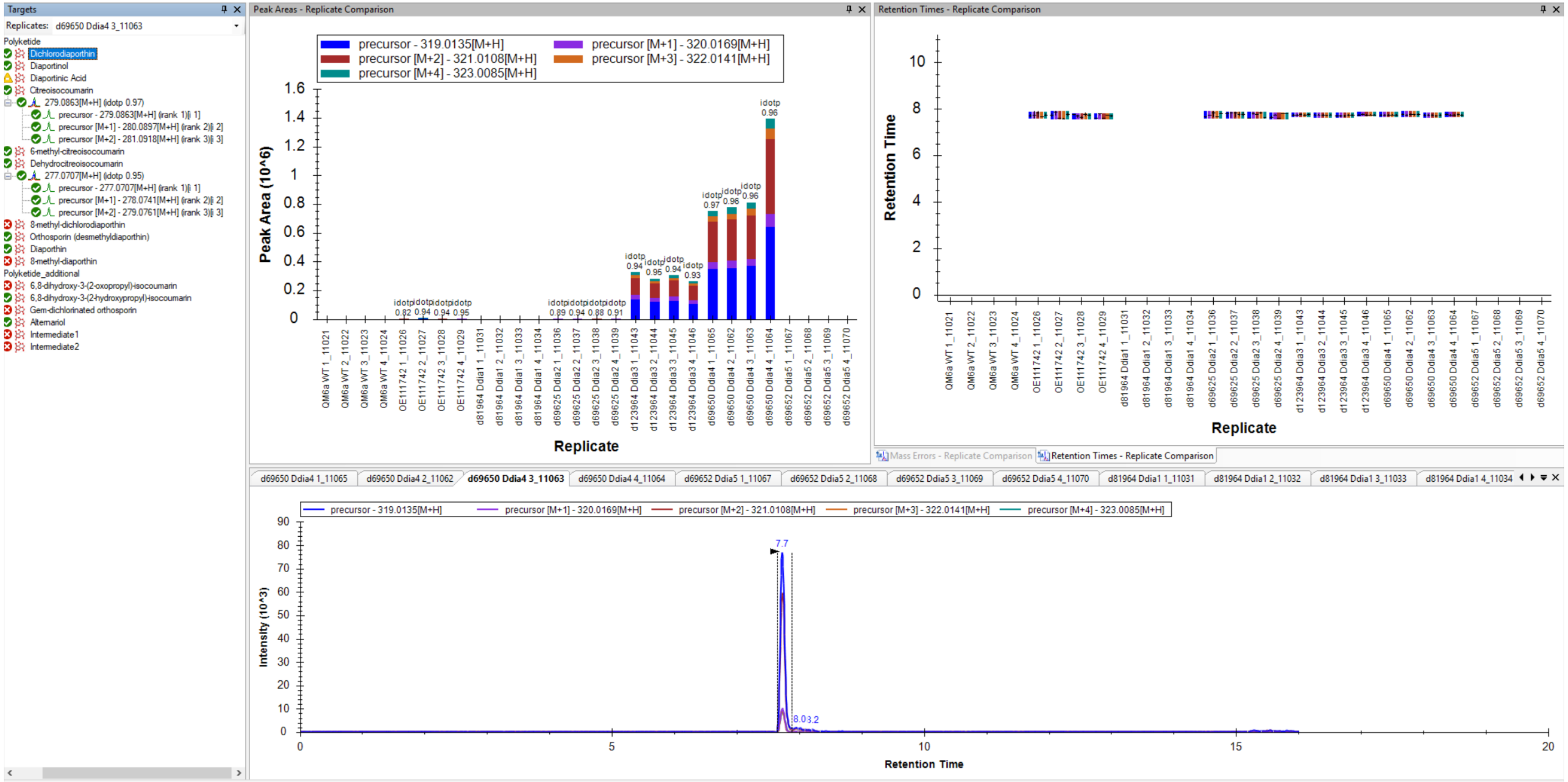

Figure S7. EIC of dichlorodiaporthin (**1**)

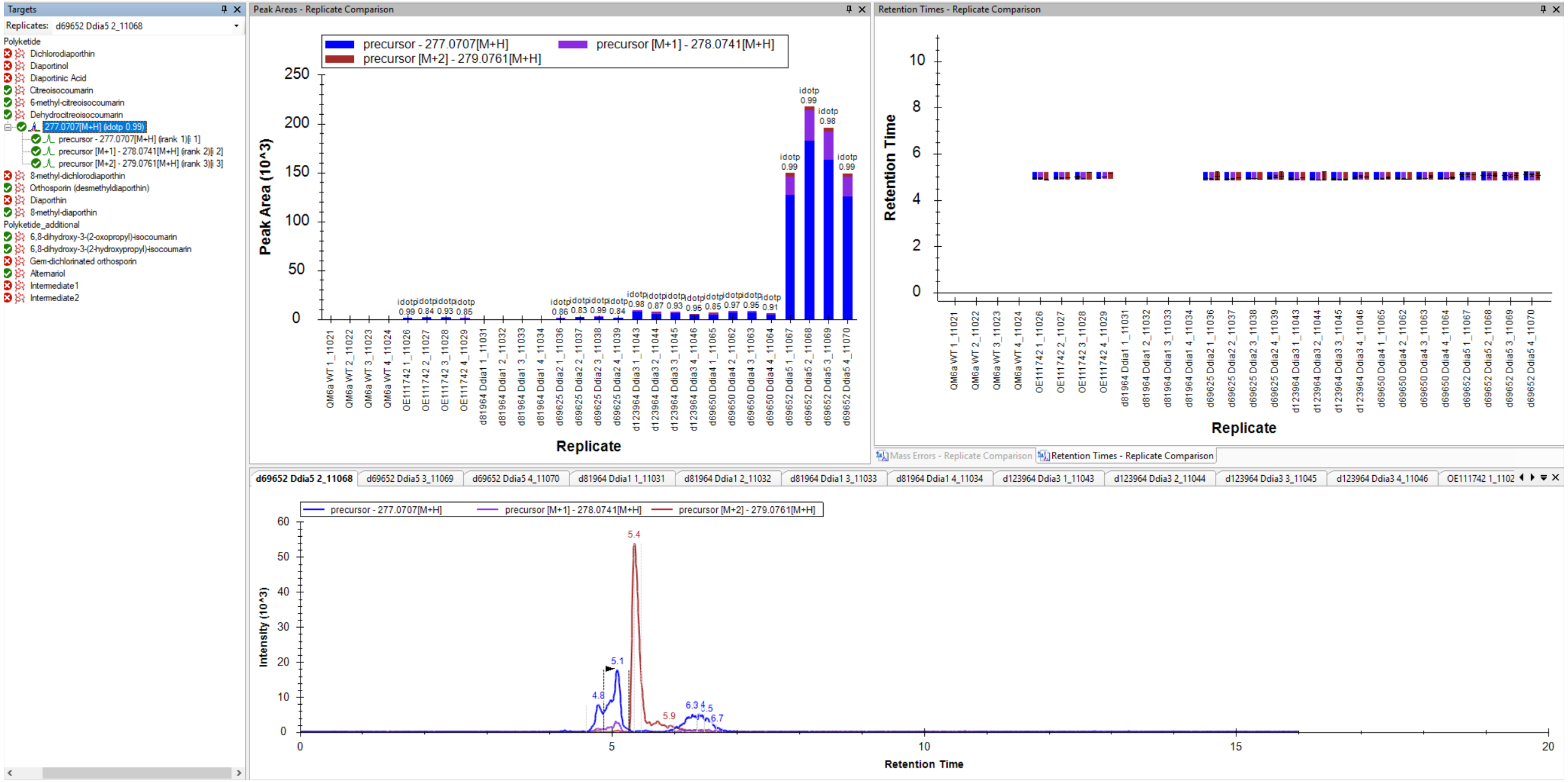

Figure S8. EIC of dehydrocitreoisocoumarin (**2**).

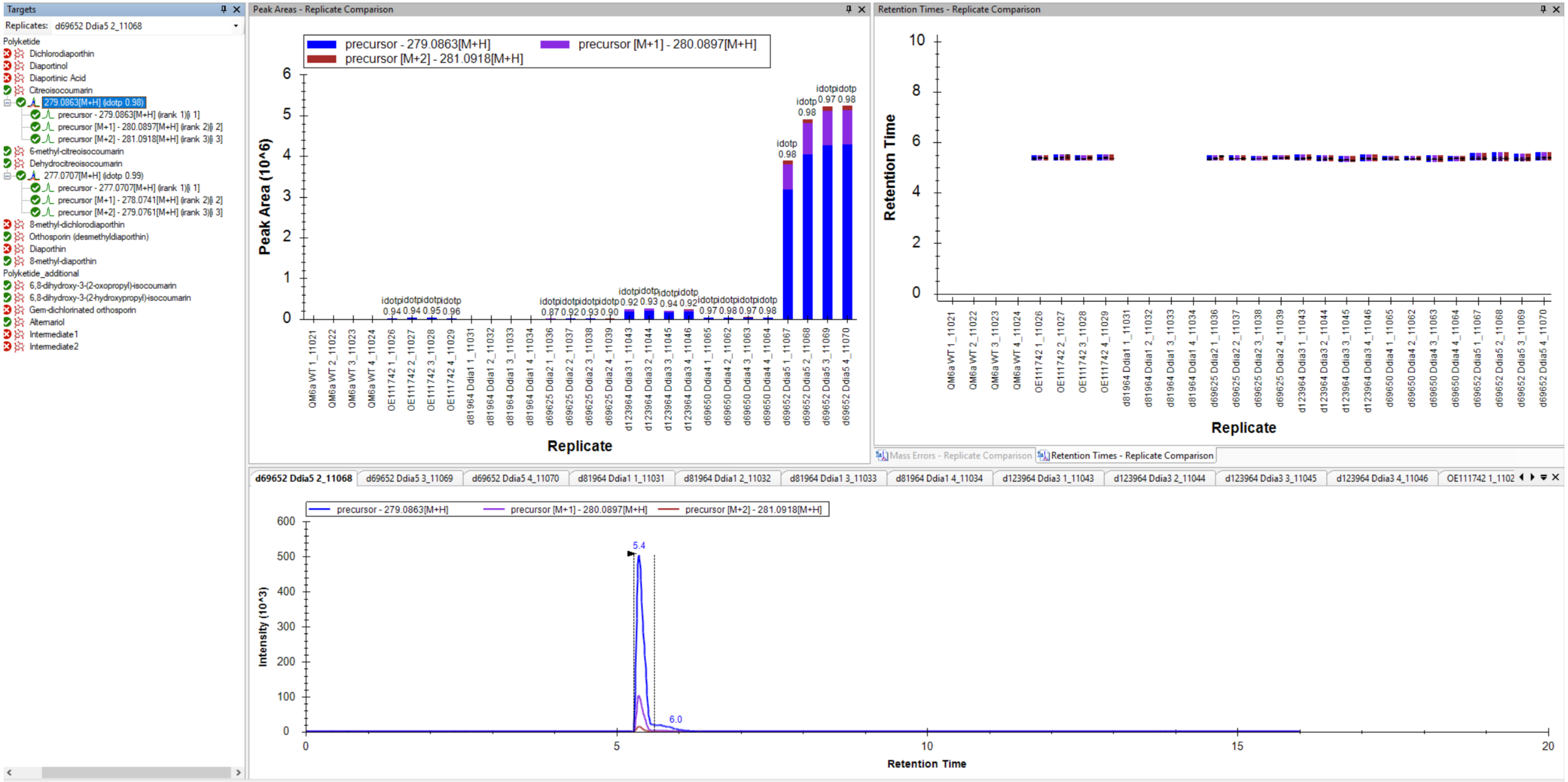

Figure S9. EIC of citreoisocoumarin (**4**).

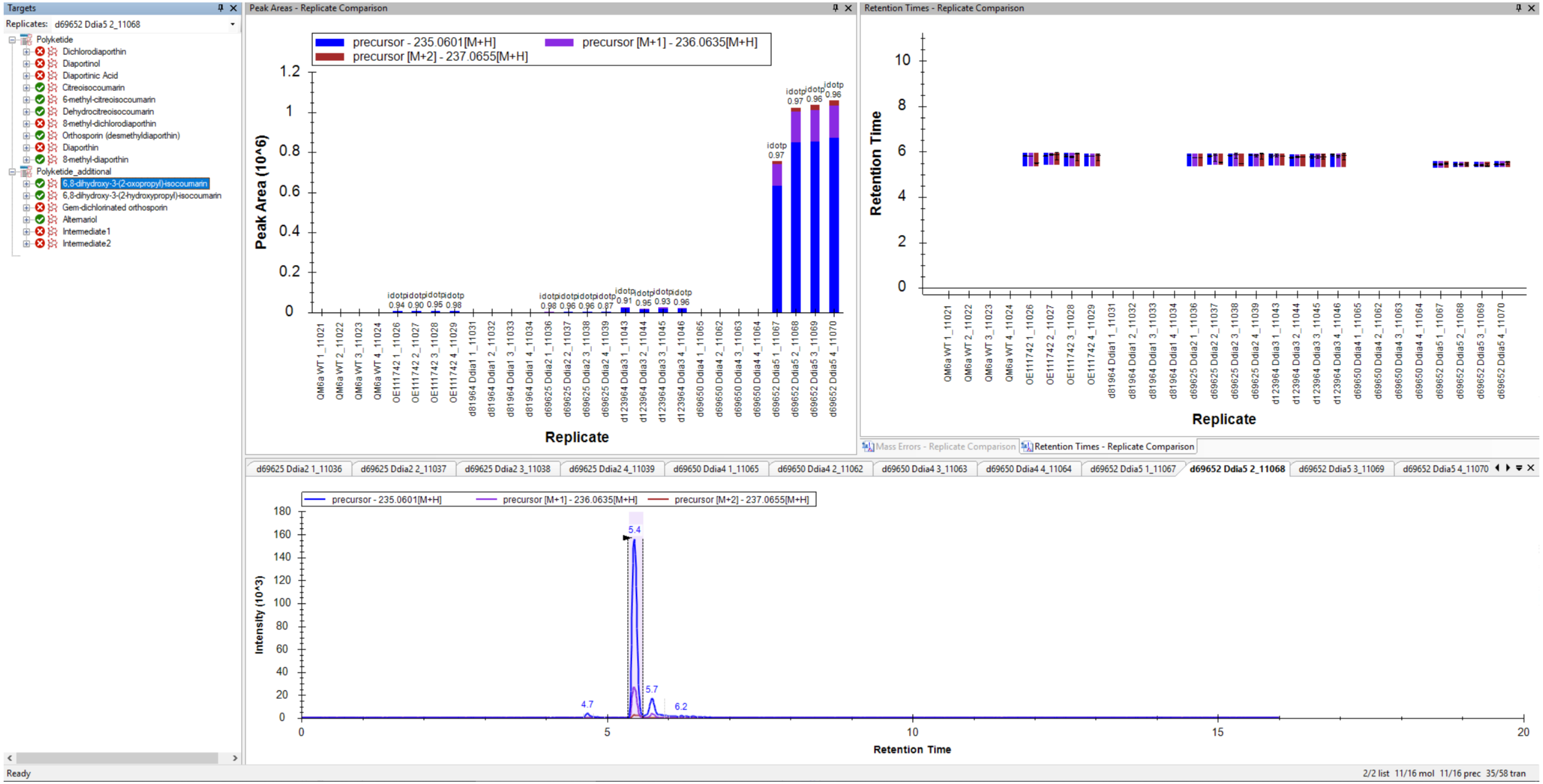

Figure S10. EIC of 6,8-dihydroxy-3-(2-oxopropyl)-isocoumarin (**6**).

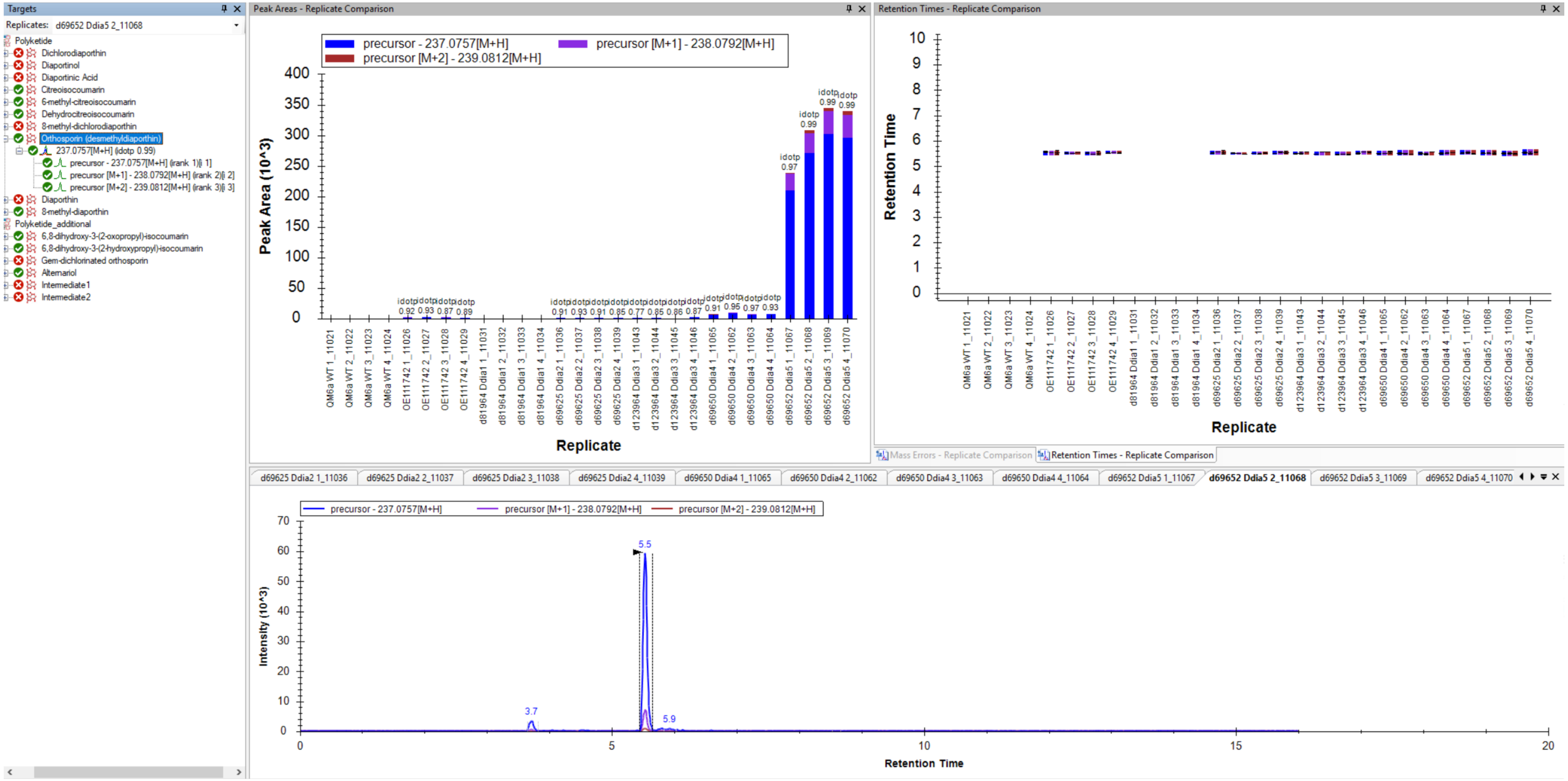

Figure S11. EIC of orthosporin (**7**).

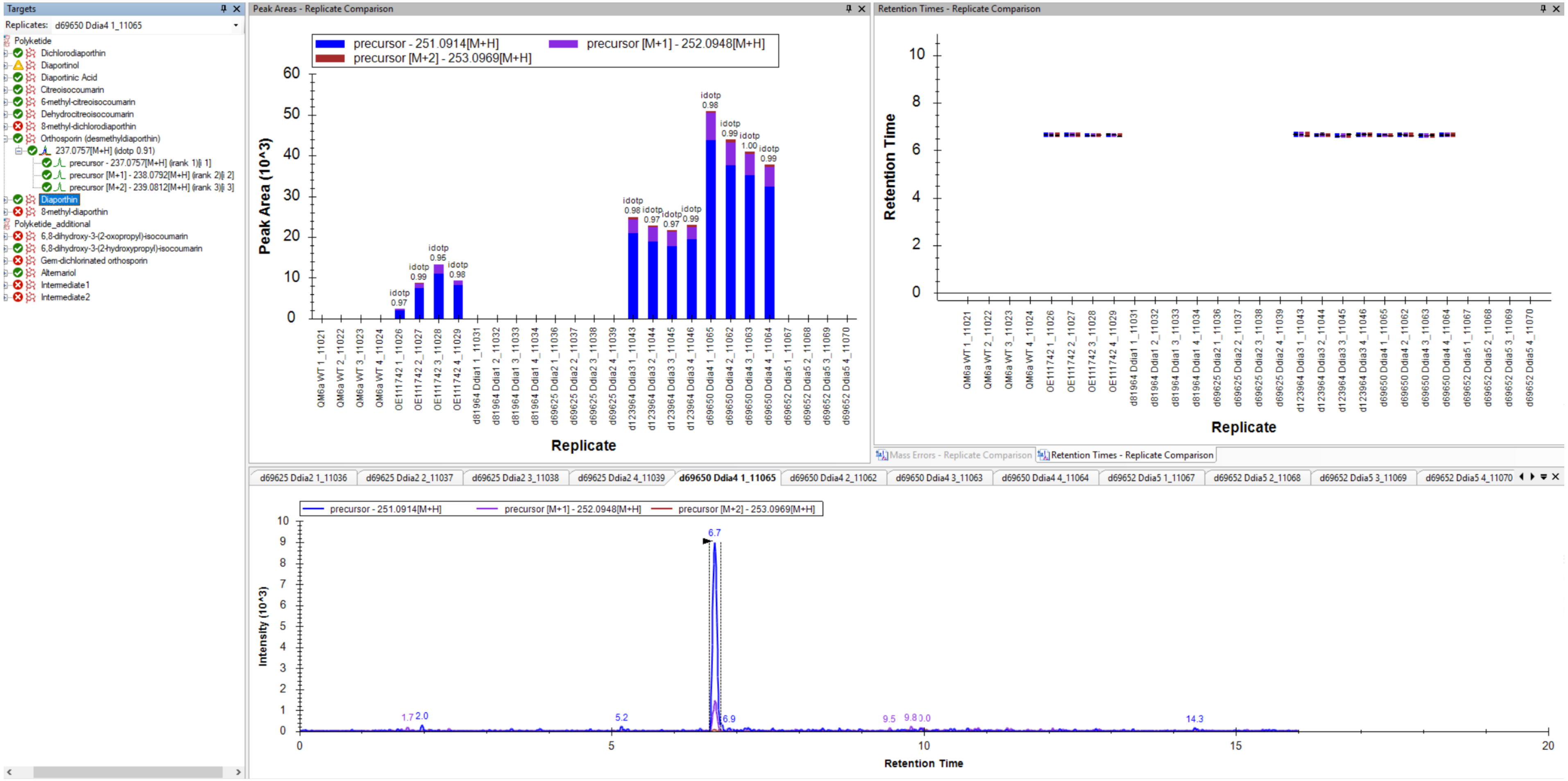

Figure S12. EIC of diaporthin (**8**).

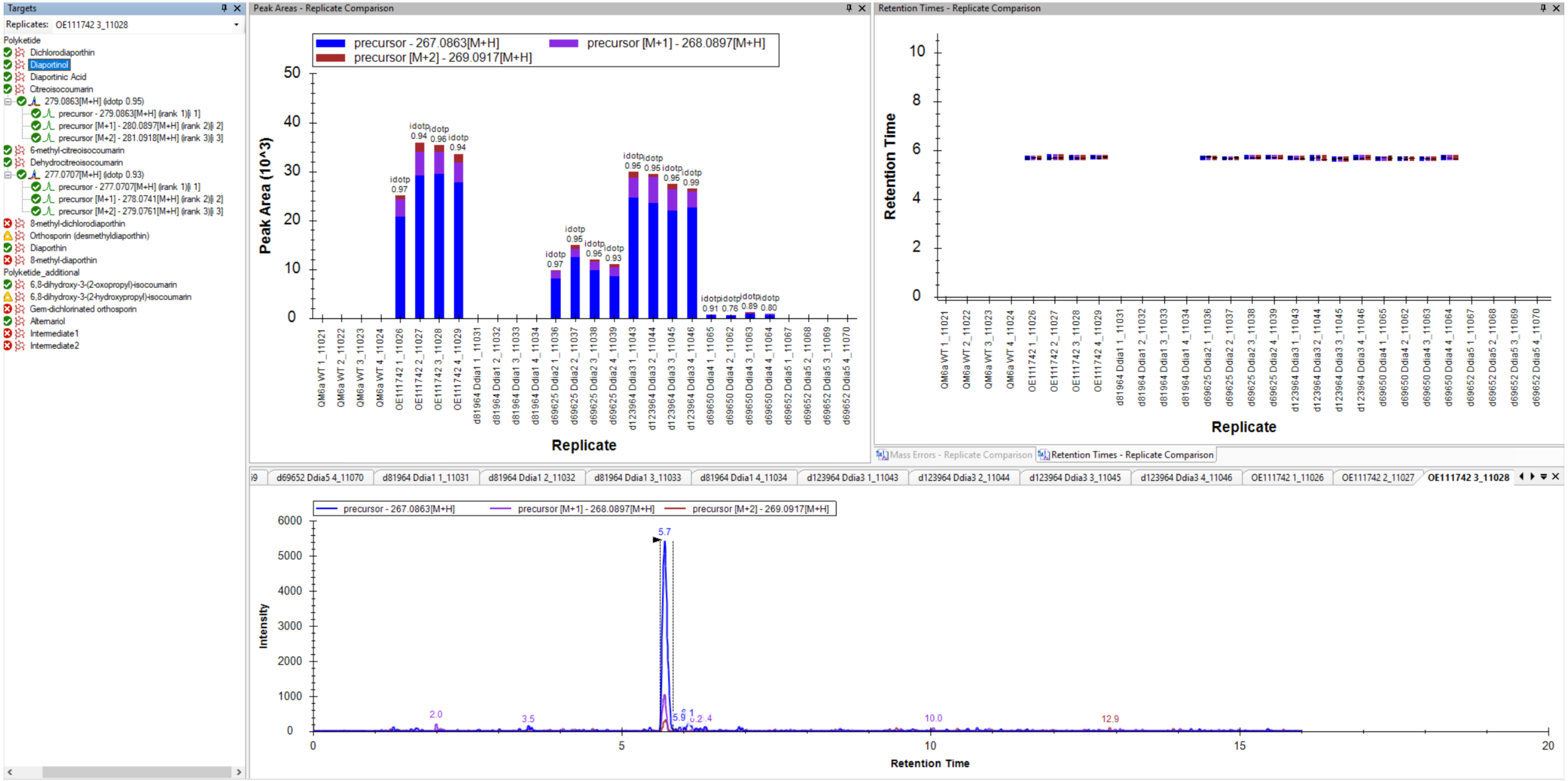

Figure S13. EIC of diaporthinol (**10**).

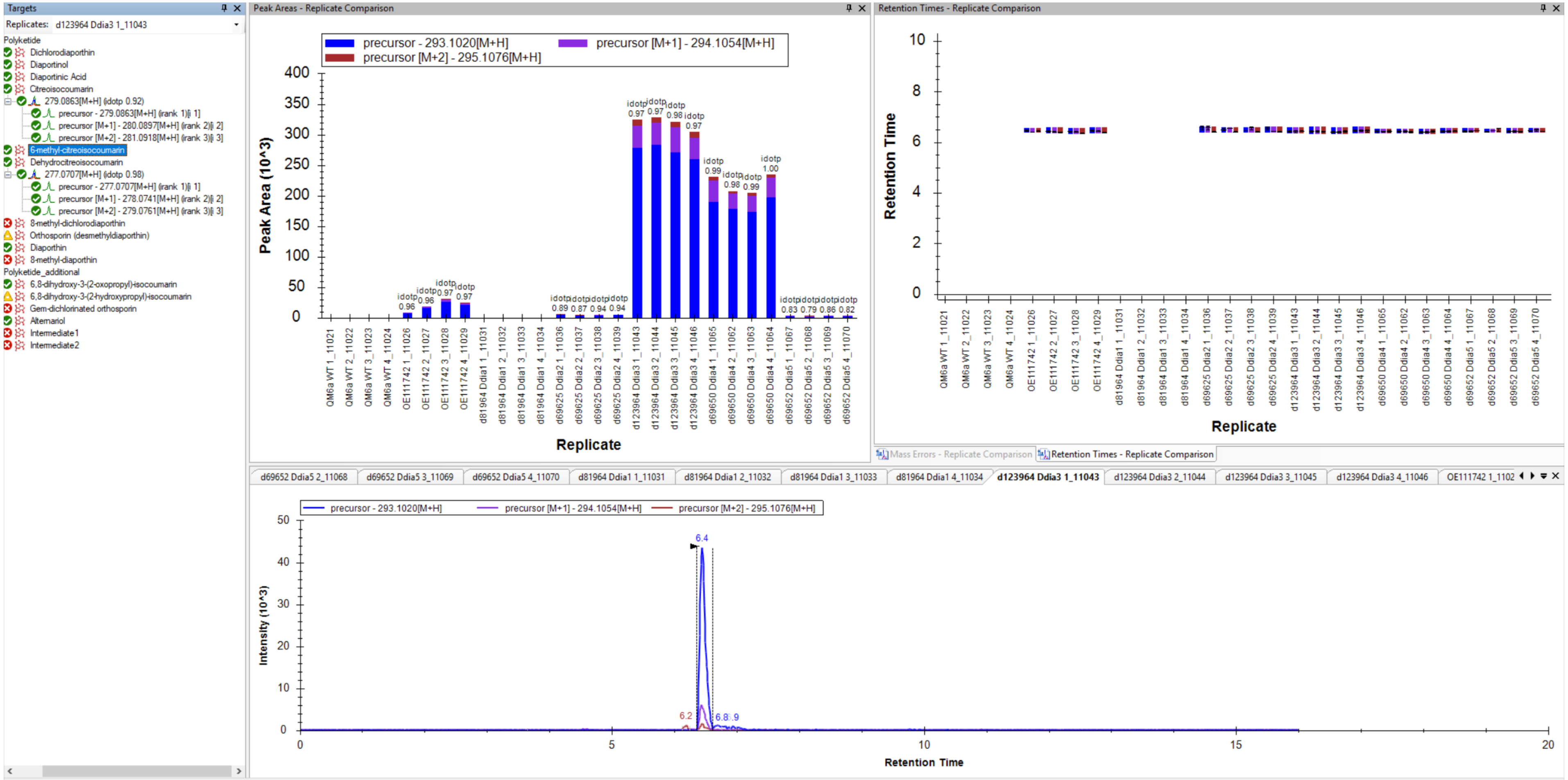

Figure S14. EIC of 6-methyl-citreoisocoumarin (**11**).

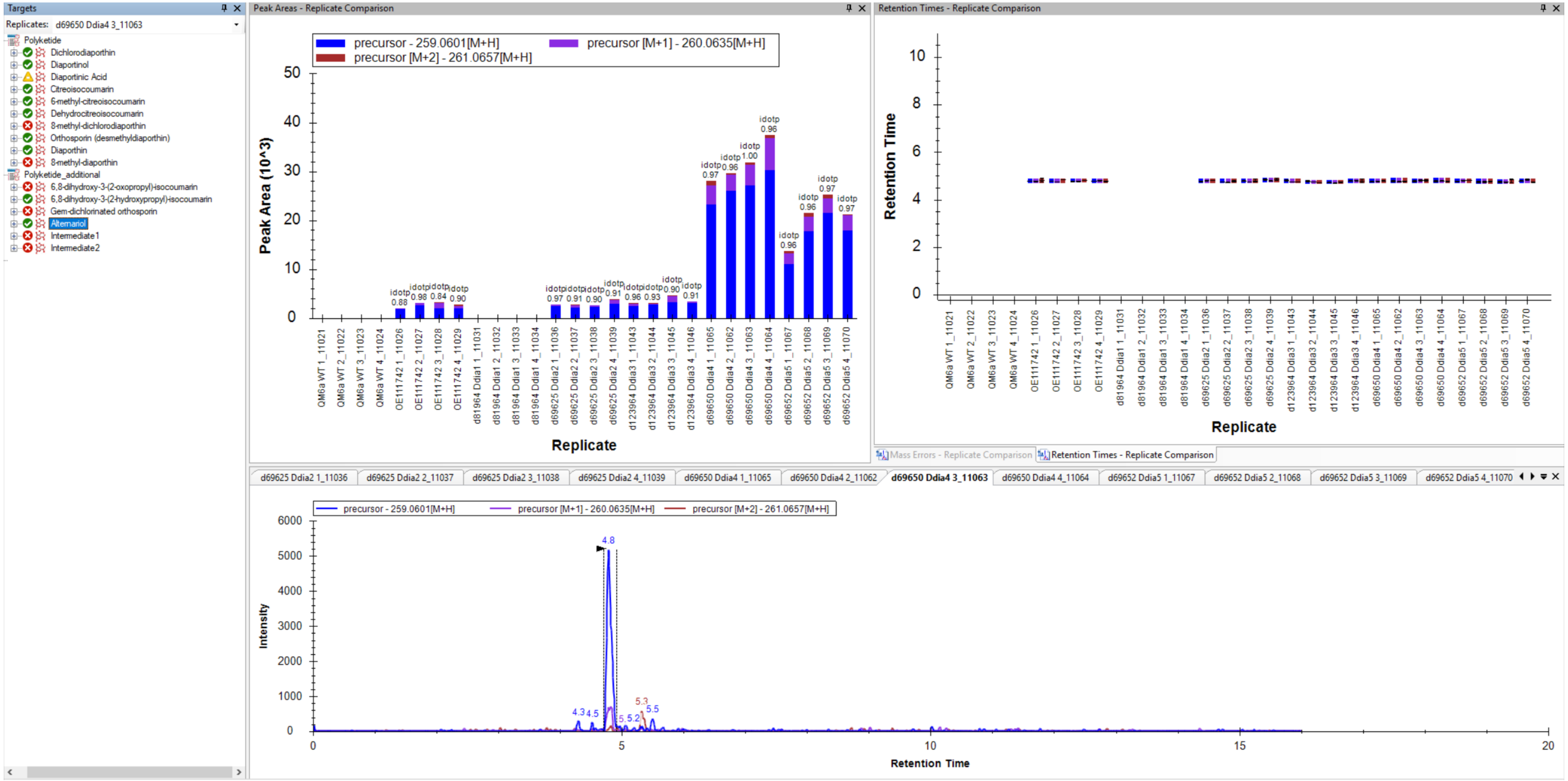

Figure S15. EIC of “not alternariol”.

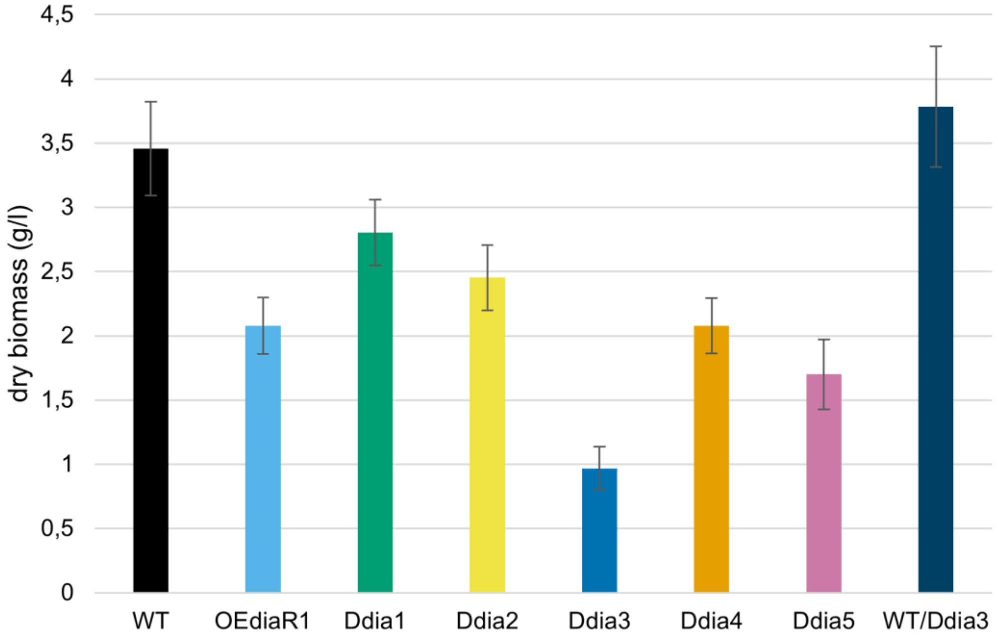

Figure S16. The indicated *T. reesei* strains were cultivated in MAM+glycerol for 48 hours and the resulting biomass harvested and dried. The measurement was performed in quadruplicates, the error bars indicate the standard deviation.

A

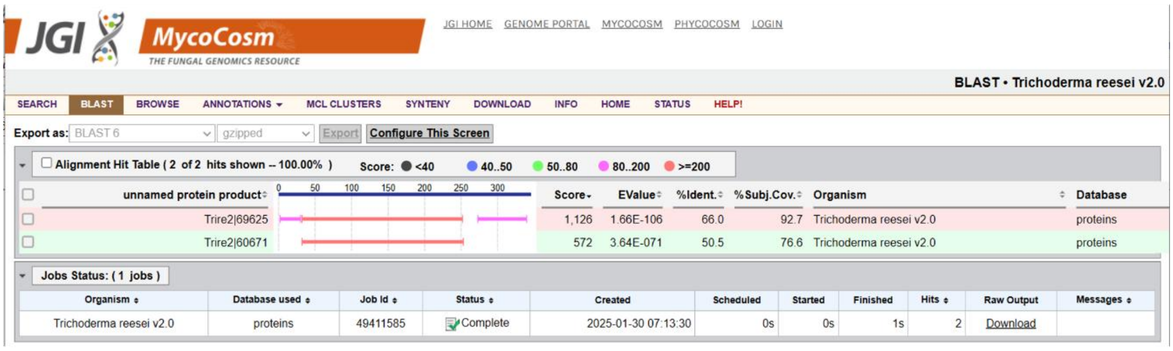

B

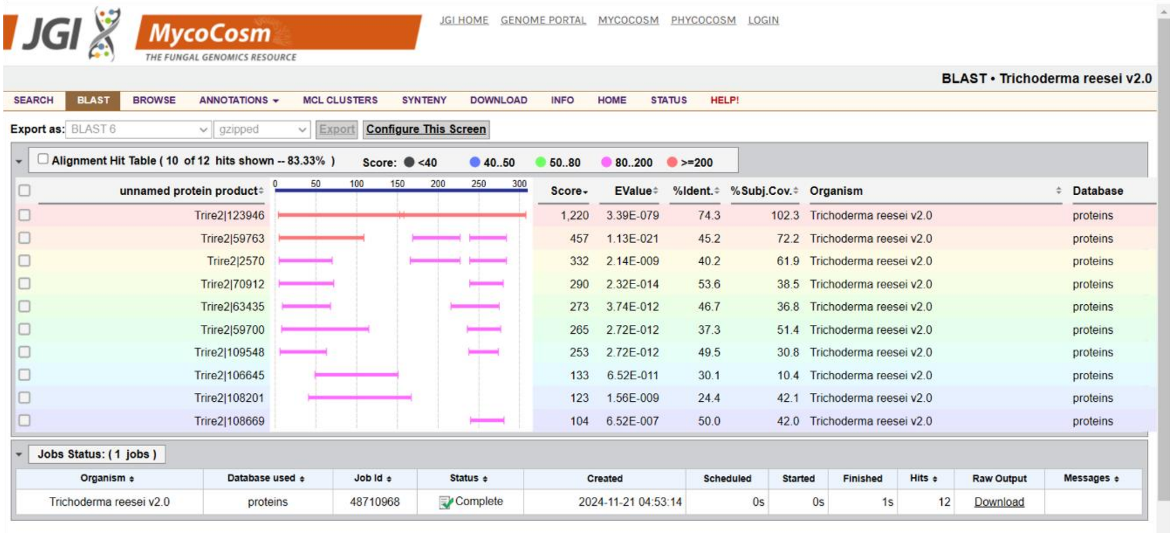

Figure S17. The protein sequences of *A. oryzae* DiaB (**A**) and DiaC (**B**) were used as query in a BLAST analysis against the proteome of *T. reesei* on https://mycocosm.jgi.doe.gov/Trire2/Trire2.home.html

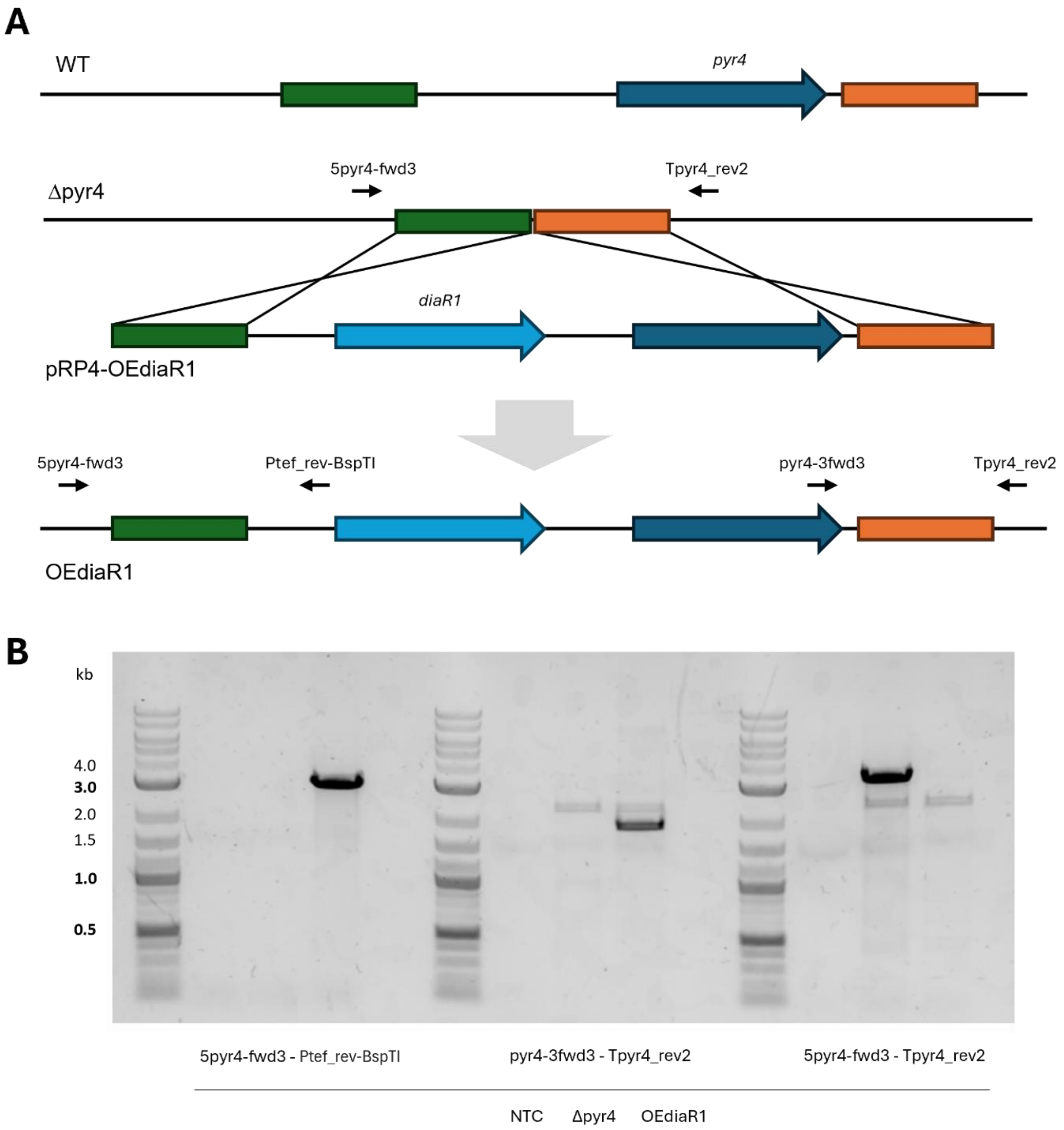

Figure S18. Construction of *T. reesei* OEdiaR1. (A) The plasmid pRP4-OEdiaR1 was linearized and inserted into *T. reesei* Δpyr4. Following a double cross-over, the expression cassette was inserted at the *pyr4* locus while simultaneously re-establishing the *pyr4* locus. The black arrows indicate the position of the primers used for genotyping. **(B)** The chromosomal DNA of the indicated strains was isolated and used as template in PCR assays using the indicated primers. NTC, no template control.

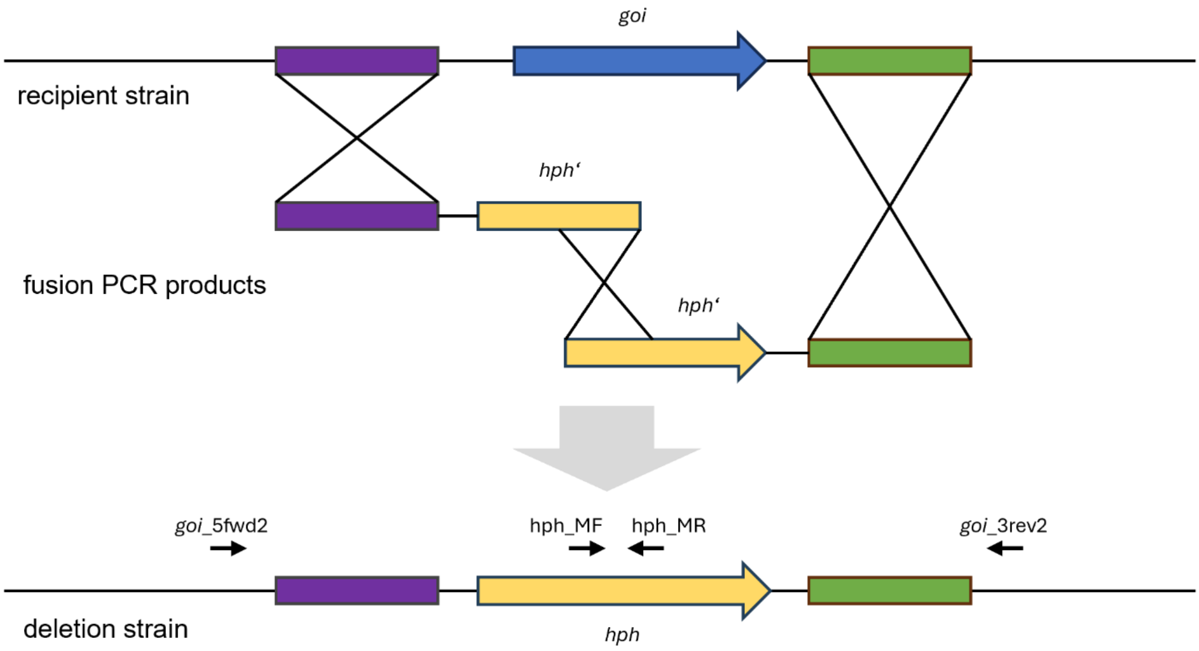

Figure S19. Split marker strategy for gene deletions. For the deletion of gene of interest (goi), two distinct fusion PCR products were constructed *in vitro* using a splicing by overlap (SOE)-PCR. The first PCR product contains the 5’flank and the first two thirds of the maker gene *hph*, while the second PCR product consists of the last 2 thirds of *hph* and the 3’flank. Upon a triple crossover, the marker is assembled and integrated at the correct locus, resulting in the deletion of the *goi* and its replacement with the *hph* marker. The black arrows indicate the positions of the primers used for genotyping in Fig. S8-S13.

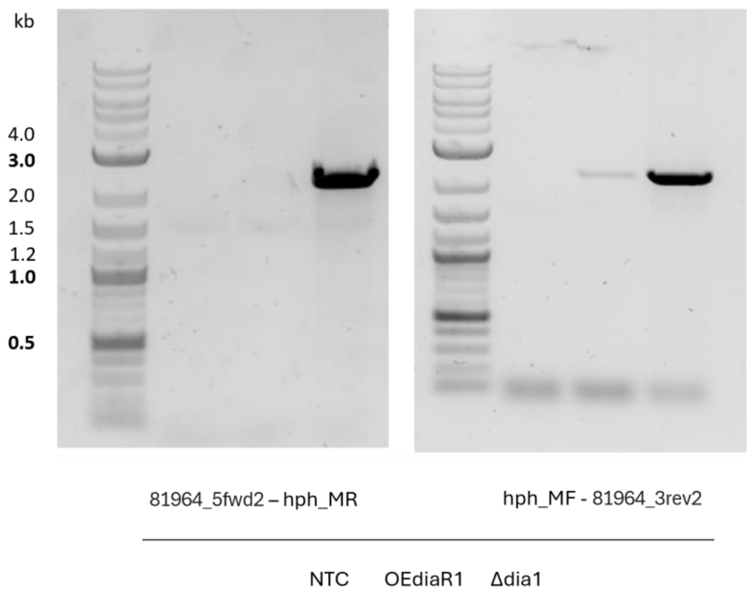

Figure S20. Genotyping of *T. reesei* Δdia1. To verify the replacement of *dia1* with the *hph* resistance cassette as depicted in Fig. S11, the chromosomal DNA of the indicated strains was isolated and used as template in PCR assays using the indicated primers. NTC, no template control.

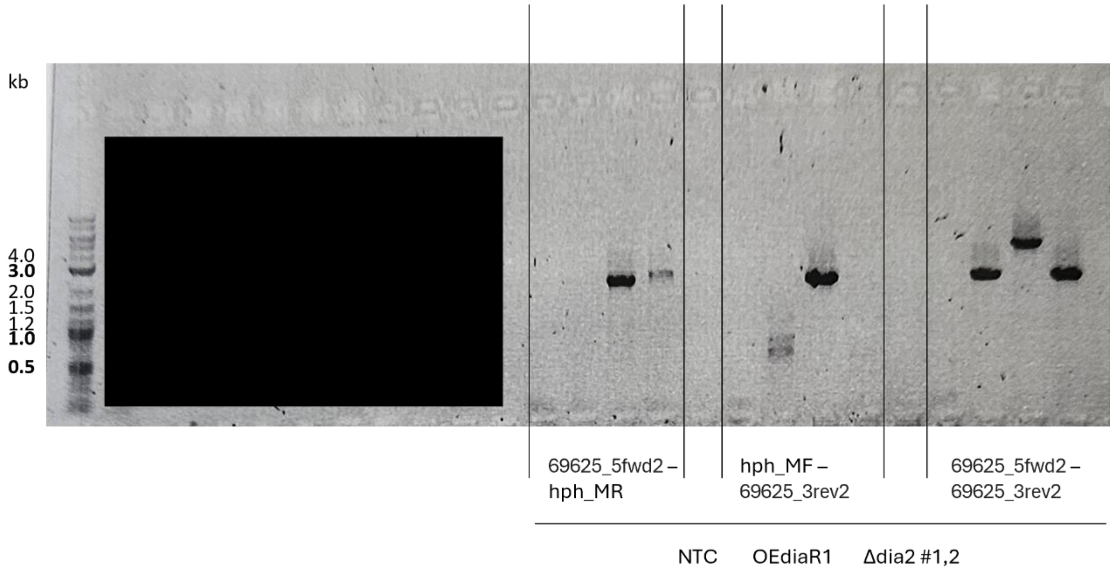

Figure S21. Genotyping of *T. reesei* Δdia2. To verify the replacement of *dia2* with the *hph* resistance cassette as depicted in Fig. S11, the chromosomal DNA of the indicated strains was isolated and used as template in PCR assays using the indicated primers. NTC, no template control.

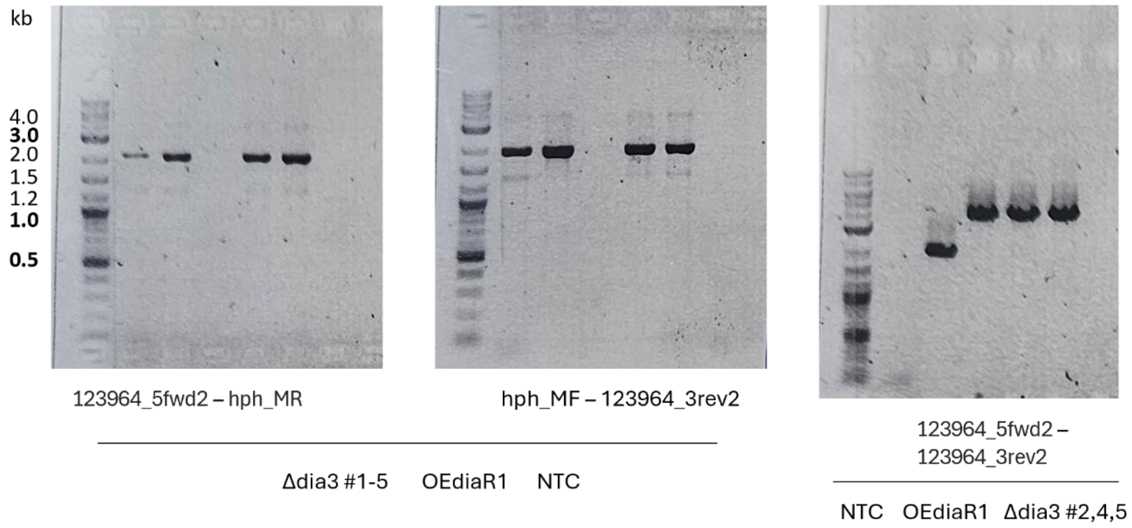

Figure S22. Genotyping of *T. reesei* Δdia3. To verify the replacement of *dia3* with the *hph* resistance cassette as depicted in Fig. S11, the chromosomal DNA of the indicated strains was isolated and used as template in PCR assays using the indicated primers. NTC, no template control.

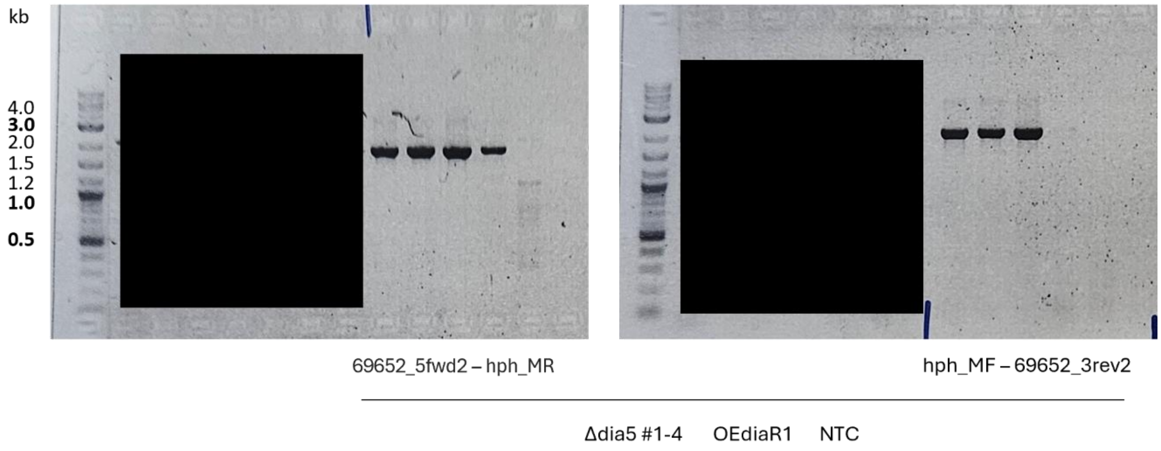

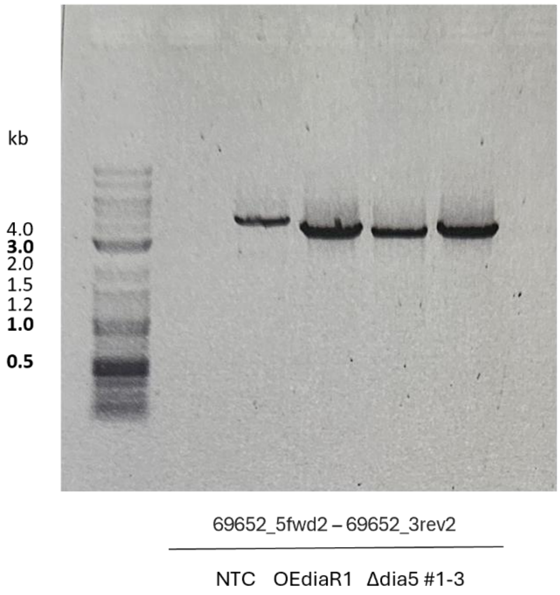

Figure S23. Genotyping of *T. reesei* Δdia5. To verify the replacement of *dia5* with the *hph* resistance cassette as depicted in Fig. S11, the chromosomal DNA of the indicated strains was isolated and used as template in PCR assays using the indicated primers. NTC, no template control.

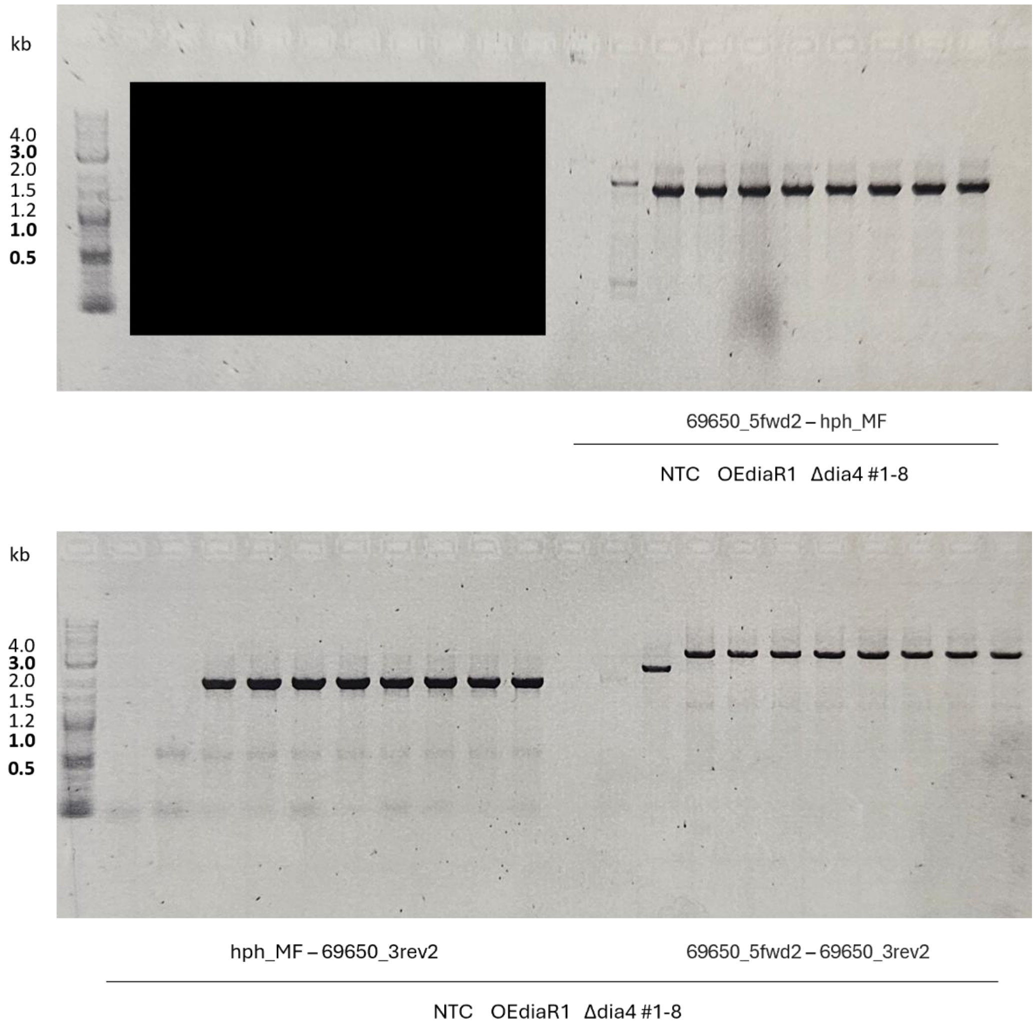

Figure S24. Genotyping of *T. reesei* Δdia4. To verify the replacement of *dia4* with the *hph* resistance cassette as depicted in Fig. S11, the chromosomal DNA of the indicated strains was isolated and used as template in PCR assays using the indicated primers. NTC, no template control.

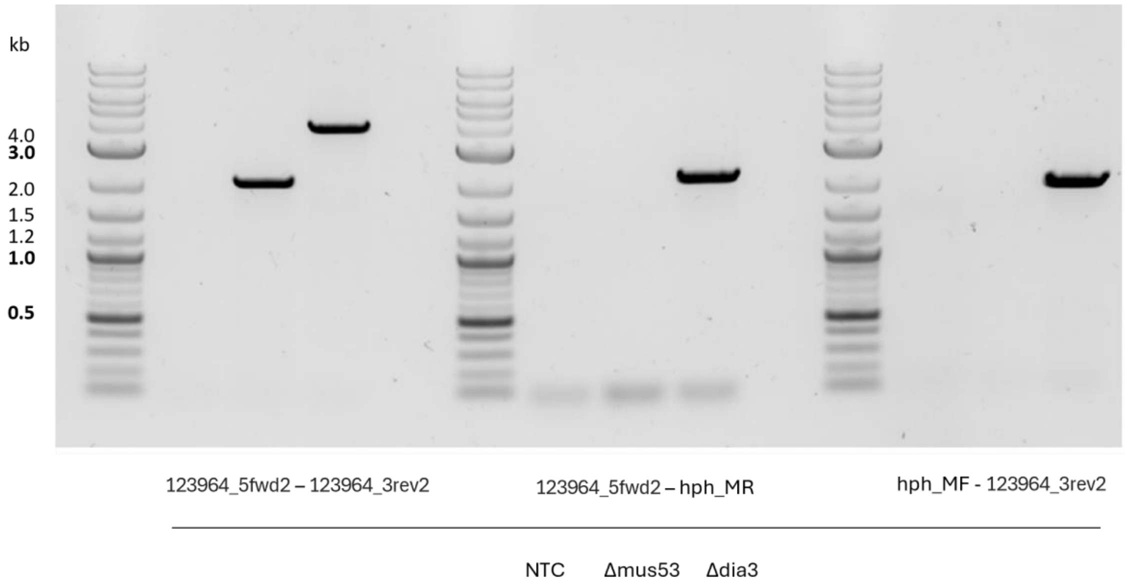

Figure S25. Genotyping of *T. reesei* WT/Δdia3. To verify the replacement of *dia3* with the *hph* resistance cassette as depicted in Fig. S11, the chromosomal DNA of the indicated strains was isolated and used as template in PCR assays using the indicated primers. NTC, no template control.

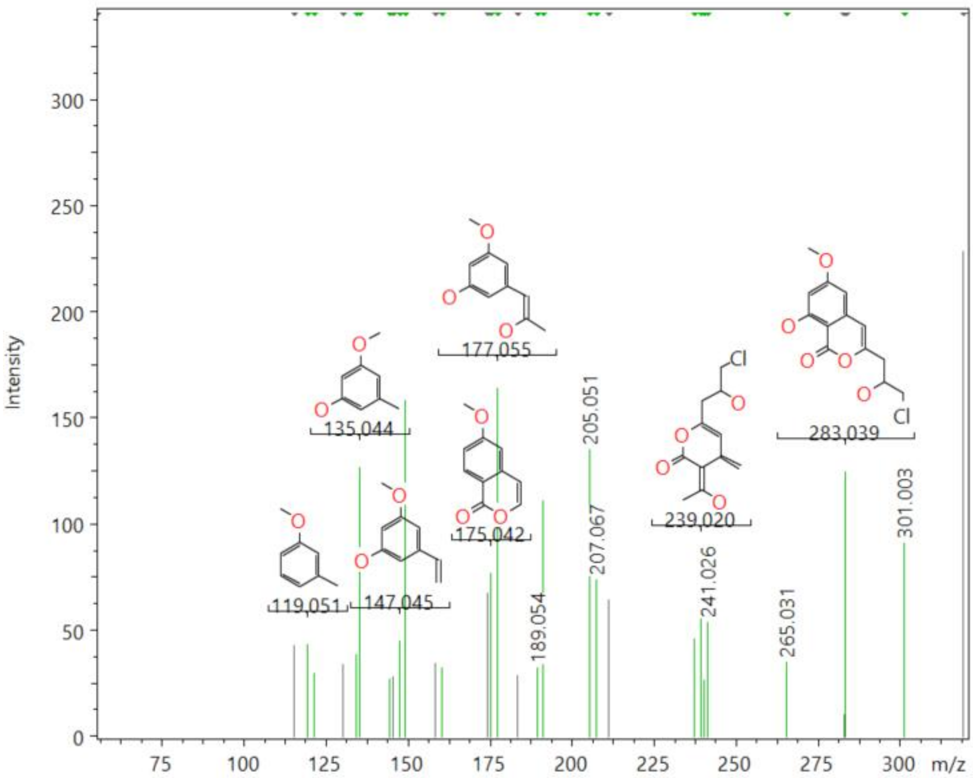

Figure S26. Comparison of obtained and *in silico* predicted fragment spectra of dichlorodiaporthin (**1**).

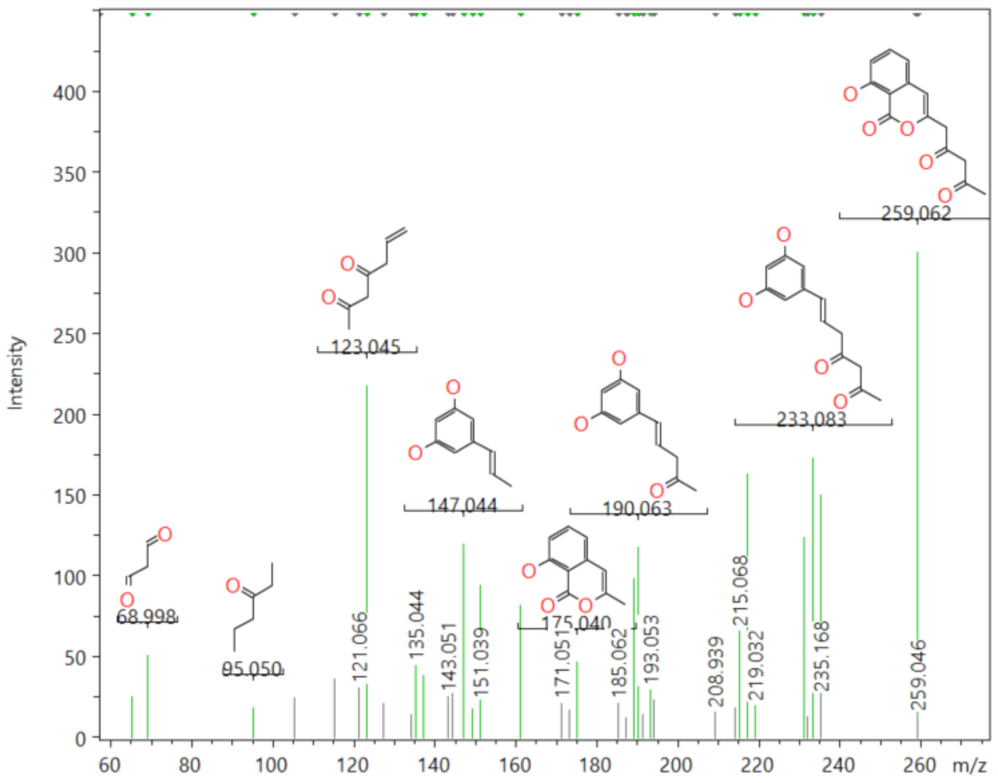

Figure S27. Comparison of obtained and *in silico* predicted fragment spectra of dehydrocitreoisocoumarin (**2**).

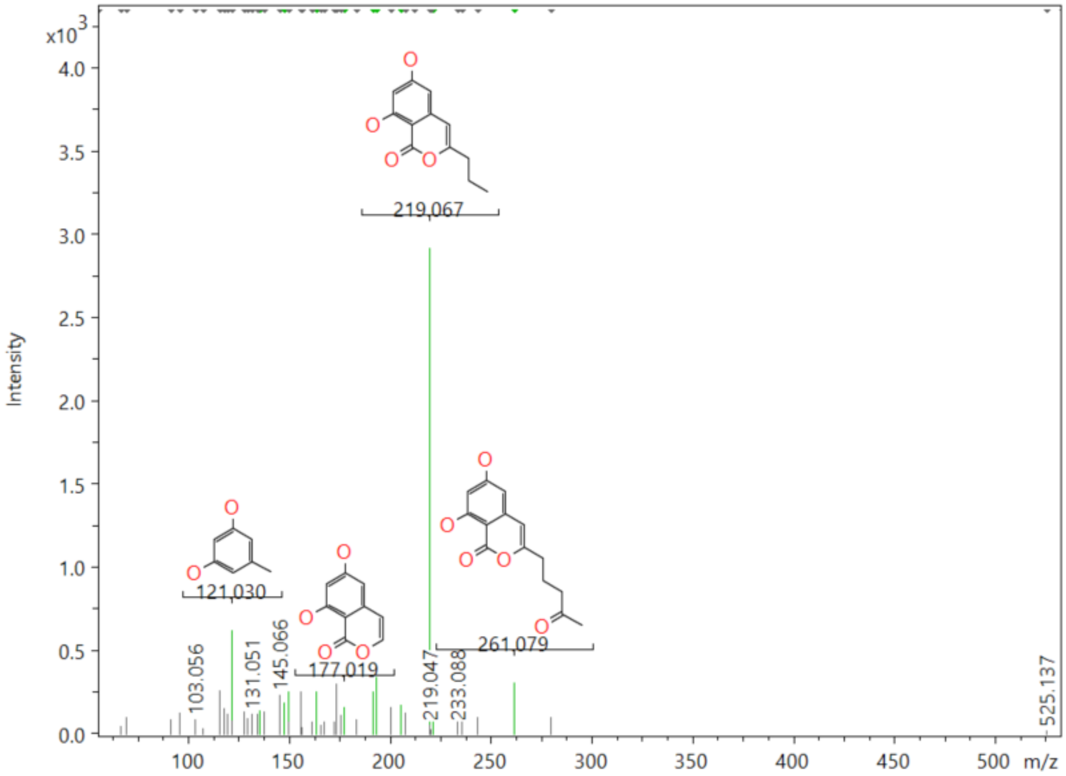

Figure S28. Comparison of obtained and *in silico* predicted fragment spectra of citreoisocoumarin (**4**).

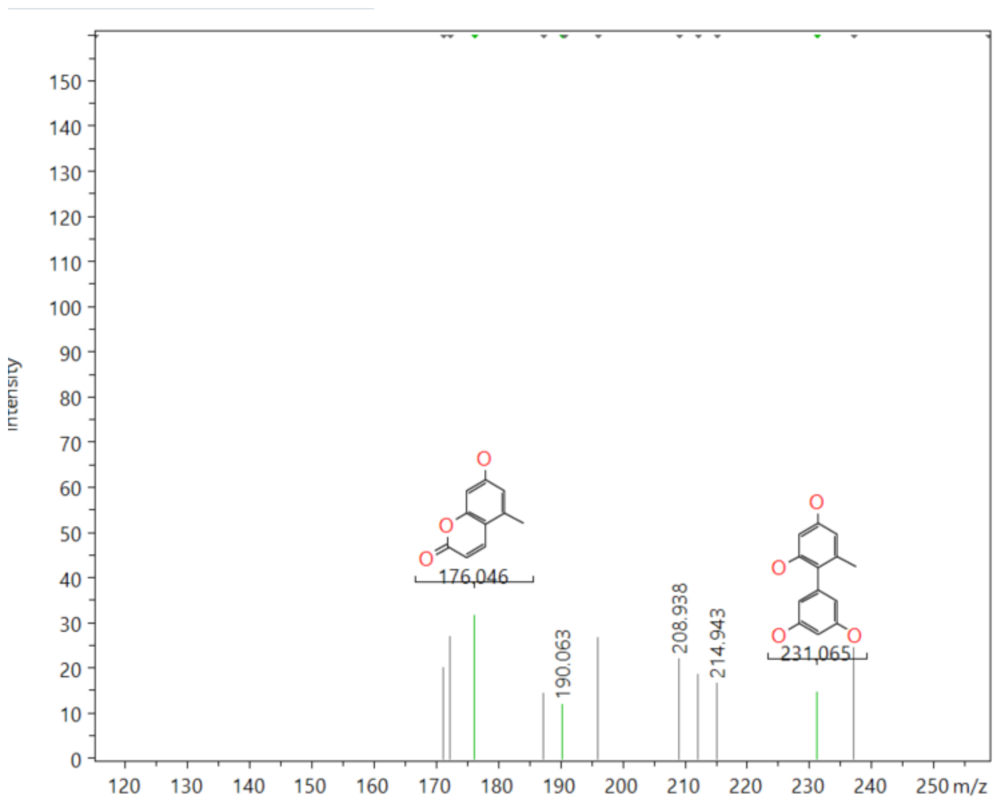

Figure S29. Comparison of obtained and *in silico* predicted fragment spectra of “not alternariol” and alternariol (**5**), respectively.

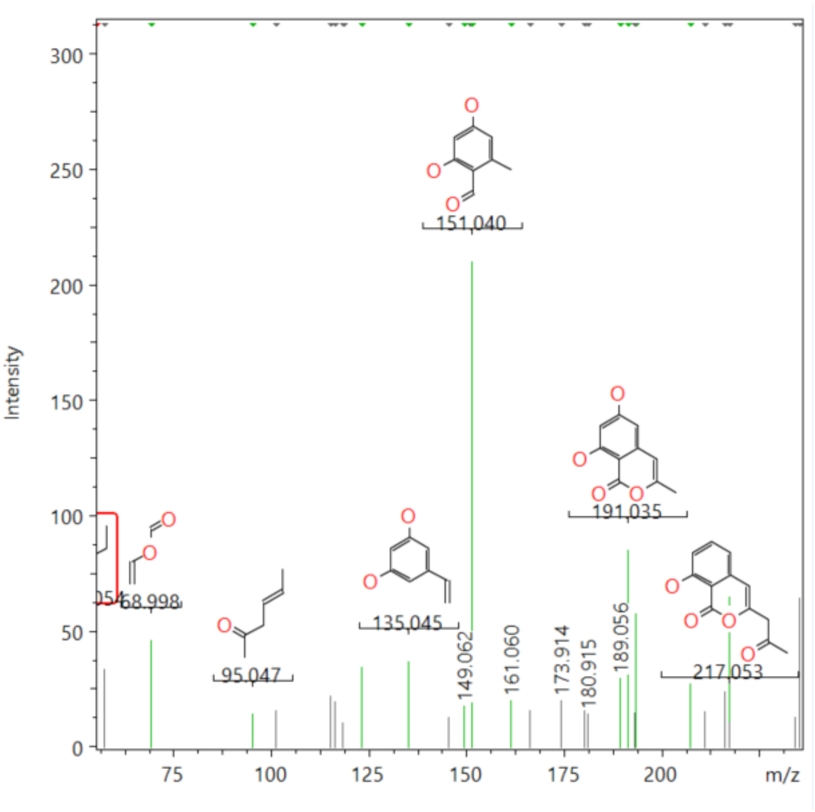

Figure S30. Comparison of obtained and *in silico* predicted fragment spectra of 6,8-dihydroxy-3-(2-oxopropyl)-isocoumarin (**6**).

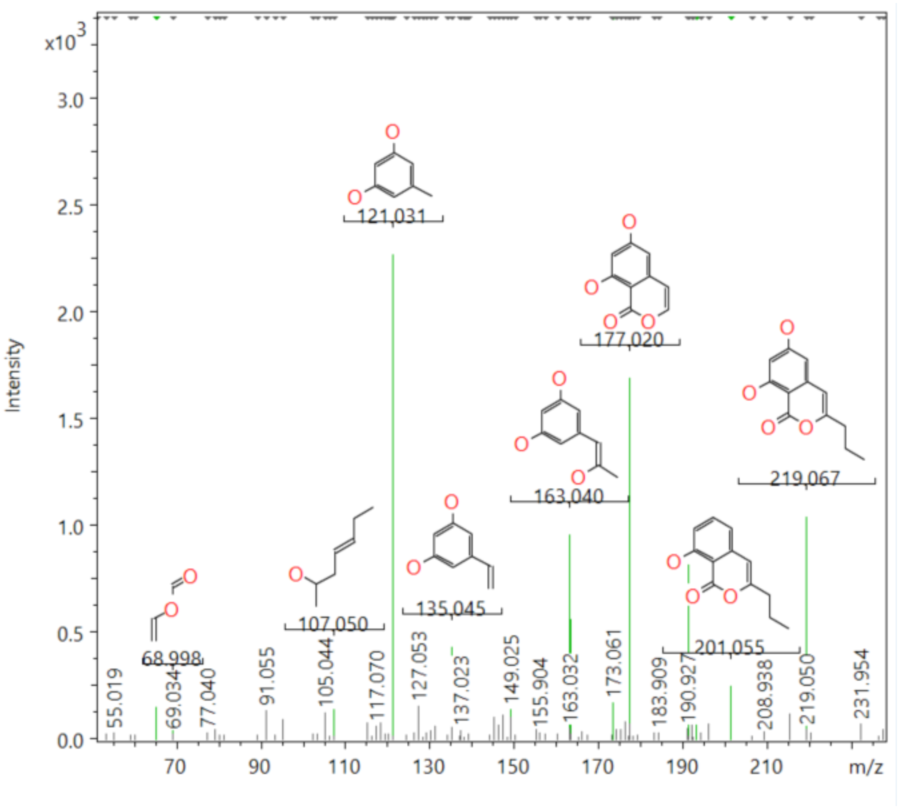

Figure S31. Comparison of obtained and *in silico* predicted fragment spectra of orthosporin (**7**).

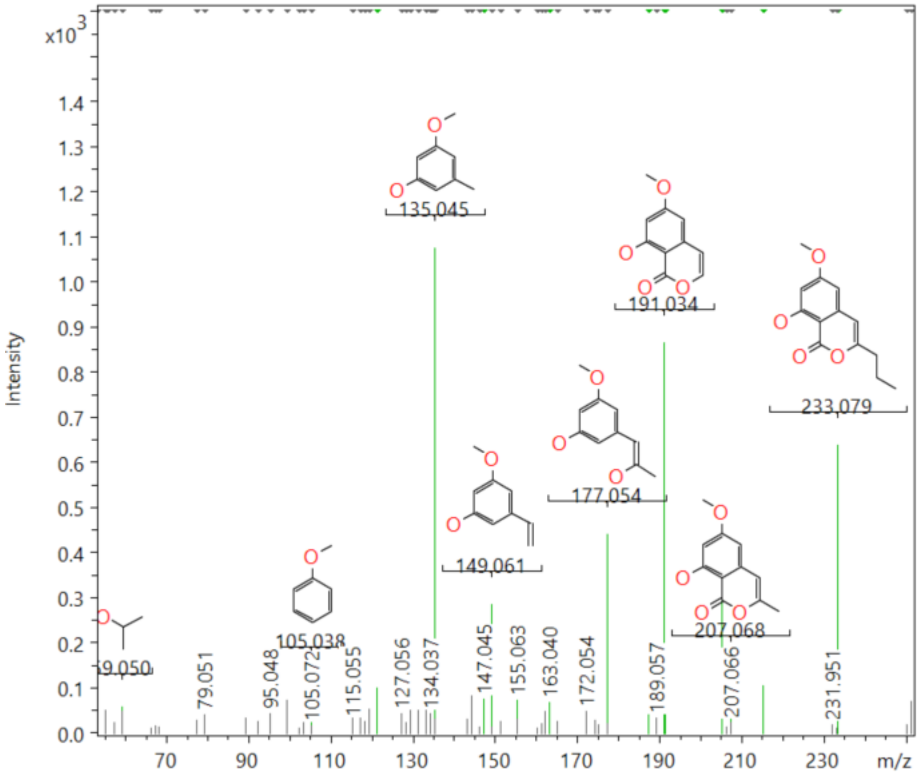

Figure S31. Comparison of obtained and *in silico* predicted fragment spectra of diaporthin (**8**).

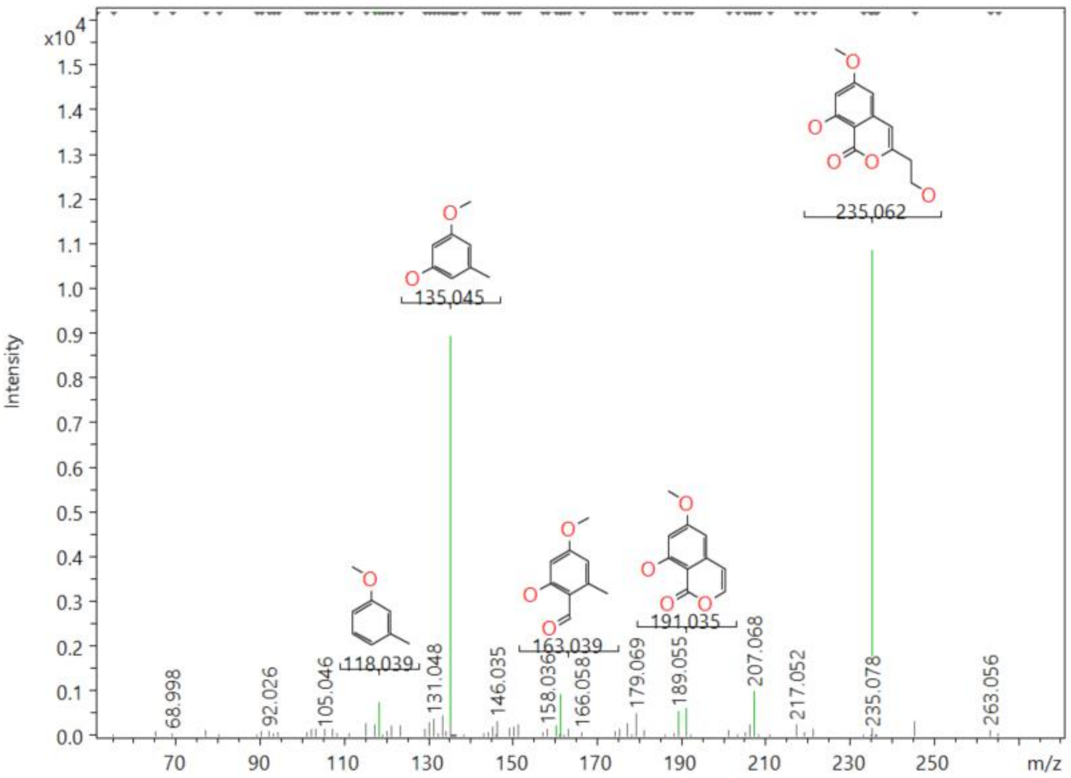

Figure S32. Comparison of obtained and *in silico* predicted fragment spectra of diaporthinic acid (**9**).

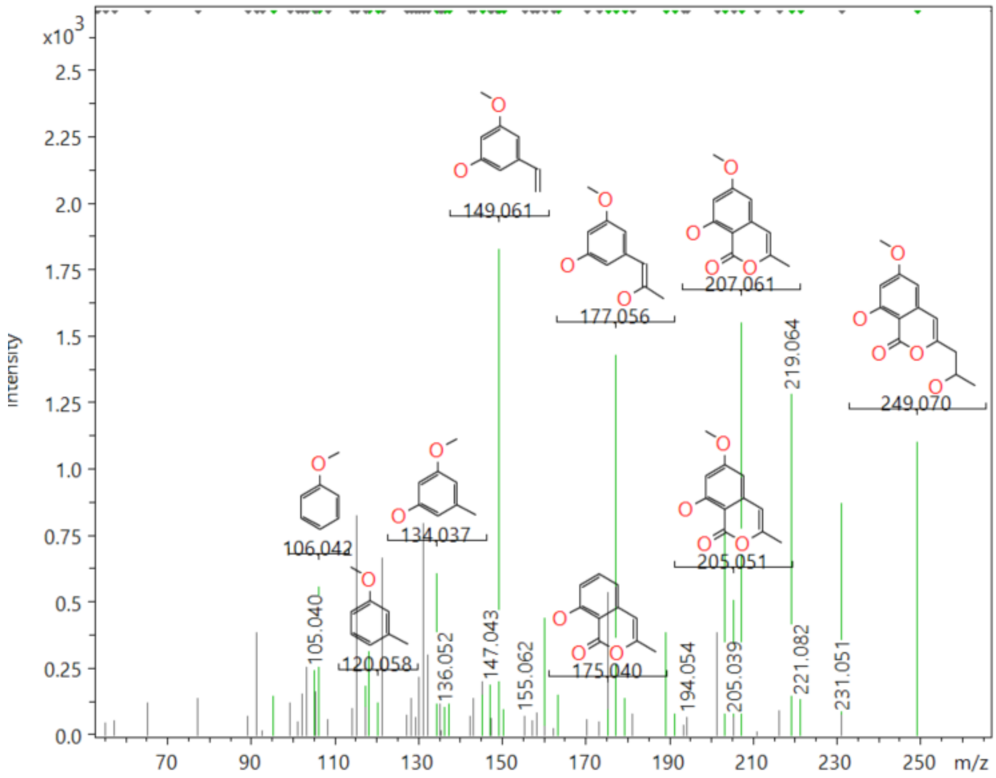

Figure S33. Comparison of obtained and *in silico* predicted fragment spectra of diaporthinol (**10**).

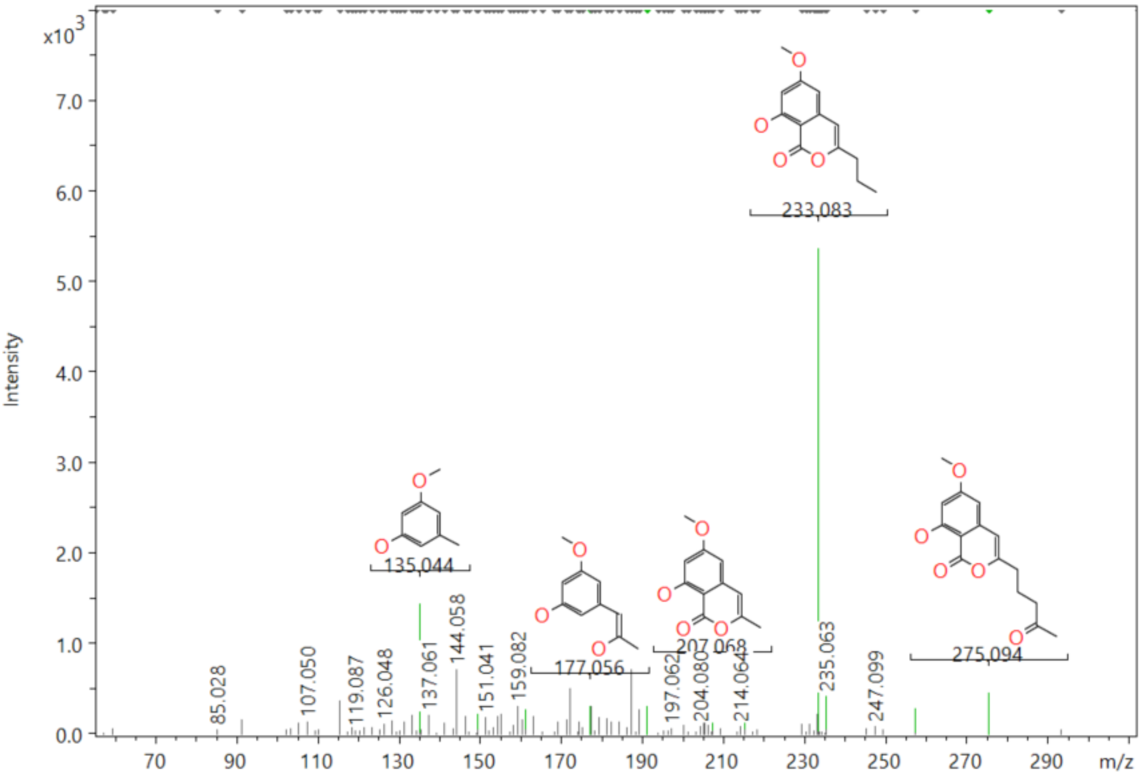

Figure S34. Comparison of obtained and *in silico* predicted fragment spectra of 6-methyl-citreoisocoumarin (**11**).

Table S1. Homologs of the *T. reesei dia* BGC genes in *D. pomorum* M27-16 on scaffold 121 (GenBank: JAKJXN020000121)

Gene name	Protein ID	Enzyme class	Homolog in *D. pomorum*
dia1	81964	polyketide synthase	KAL1641334
dia2	69625	beta-lactamase-like	JAKJXN020000121 (22,623 – 23,120)
dia3	123964	dehydrogenase	KAL1641336
dia5	69652	bifunctional flavin-dependent halogenase / methyltransferase	KAL1641335
dia4	69650	the FAD-dependent oxioreductase	KAL1641337
diaR1	111742	zinc cluster protein	JAKJXN020000121 (15,791 - 14,892)
